# Excavating the functionally crucial active-site residues of the DXS protein of *Bacillus subtilis* by exploring its closest homologues

**DOI:** 10.1186/s43141-020-00087-x

**Published:** 2020-11-26

**Authors:** Ashish Runthala, Tavakala Harsha Sai, Vandana Kamjula, Suresh C. Phulara, Vikrant Singh Rajput, Karthikeyan Sangapillai

**Affiliations:** 1 Koneru Lakshmaiah Education Foundation, Guntur, 522502 India; 2 School of Biotechnology, Jawaharlal Nehru University, New Delhi, India

**Keywords:** DXS, Motif, Phylogeny, Consurf, Coevolution, Directed evolution

## Abstract

**Background:**

To achieve a high yield of terpenoid-based therapeutics, 1-deoxy-d-xylulose-5-phosphate (DXP) pathway has been significantly exploited for the production of downstream enzymes. The DXP synthase (DXS) enzyme, the initiator of this pathway, is pivotal for the convergence of carbon flux, and is computationally studied well for the industrially utilized generally regarded as safe (GRAS) bacterium *Bacillus subtilis* to decode its vital regions for aiding the construction of a functionally improved mutant library.

**Results:**

For the 546 sequence dataset of DXS sequences, a representative set of 108 sequences is created, and it shows a significant evolutionary divergence across different species clubbed into 37 clades, whereas three clades are observed for the 76 sequence dataset of *Bacillus subtilis*. The DXS enzyme, sharing a statistically significant homology to transketolase, is shown to be evolutionarily too distant. By the mutual information-based co-evolutionary network and hotspot analysis, the most crucial loci within the active site are deciphered. The 650-residue representative structure displays a complete conservation of 114 loci, and only two co-evolving residues ASP154 and ILE371 are found to be the conserved ones. Lastly, P318D is predicted to be the top-ranked mutation causing the increase in the thermodynamic stability of 6OUW.

**Conclusion:**

The study excavates the vital functional, phylogenetic, and conserved residues across the active site of the DXS protein, the key rate-limiting controller of the entire pathway. It would aid to computationally understand the evolutionary landscape of this industrially useful enzyme and would allow us to widen its substrate repertoire to increase the enzymatic yield of unnatural molecules for in vivo and in vitro applications.

**Supplementary Information:**

The online version contains supplementary material available at 10.1186/s43141-020-00087-x.

## Introduction

**Fig. 1:**
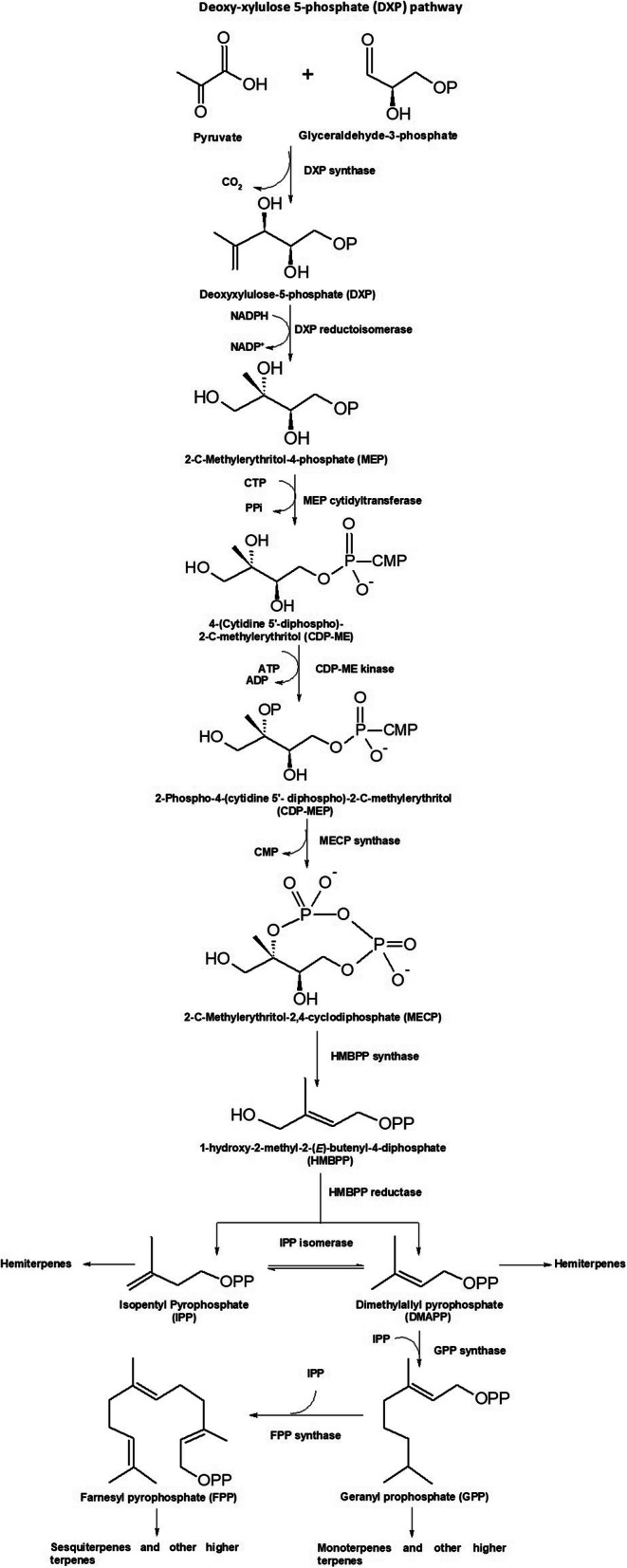
The MEP pathway. The pathway produces IPP and DMAPP as the 5-carbon building blocks for the biosynthesis of isoprenoids. It condenses glyceraldehyde 3-phopshate and pyruvate through DXS and forms IPP and DMAPP through six added steps. DMAPP linkage with one or two molecules of IPP forms monoterpene or sesquiterpene, respectively

**Fig. 2:**
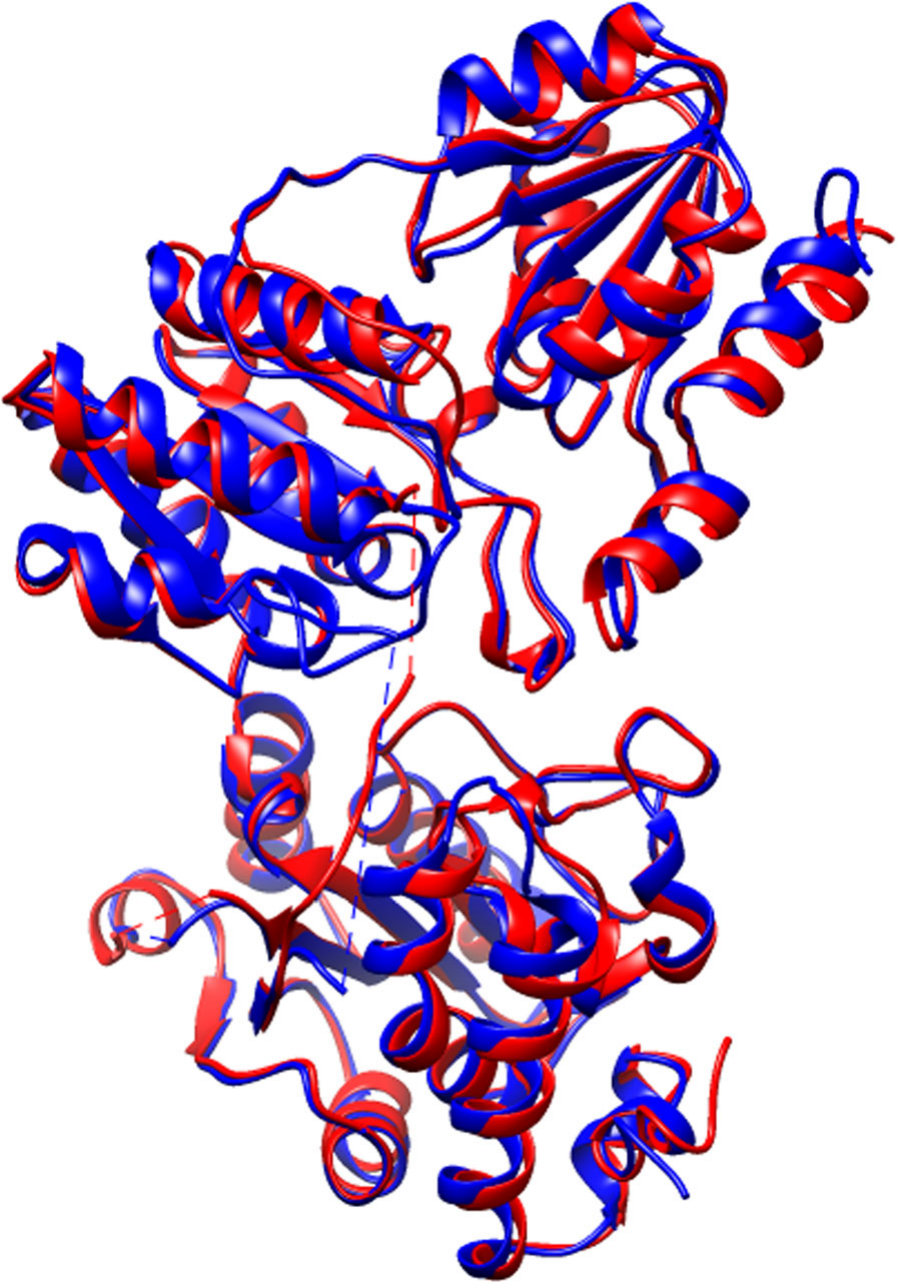
Structural overlap of DXS structures of *Escherichia coli* and *Deinococcus radiodurans*, showing a structural conservation of 468 residues across the secondary structure elements. The structures are superimposed and represented through Chimera 1.14 [21]

Isoprenoids constitute the largest class of structurally and functionally diverse secondary metabolites and encompass more than 55,000 known compounds [1, 2]. These compounds have been traditionally deployed for the synthesis of aromatic, flavoring, and pharmaceutical molecules [3–8]. To date, plants are the major source of isoprenoid based bioactive molecules [9, 10], and it has led to an overexploitation of plants, causing severe environmental issues. For example, due to the heavy exploitation of *Taxus wallichiana* (Himalayan Yew) for the extraction of pharmacologically important isoprenoids [11], a 90% decline has been reported in its population across the Indo-Nepal Himalayan region and is, therefore, declared as an endangered species by the international union for conservation of nature (IUCN) [12]. Therefore, the global interest has now shifted to produce the isoprenoid based bioactive molecules from generally regarded as safe (GRAS) status microbes, such as *Bacillus subtilis* for pharmaceutical and nutraceutical applications [13, 14]. *Bacillus subtilis* produces isoprenoid compounds via 1-deoxy-d-xylulose-5-phosphate (DXP) pathway which recruits seven enzymatic steps for the conversion of glyceraldehyde 3-phosphate (G3P) and pyruvate into prenyl precursors: Dimethylallyl diphosphate (DMAPP) and isopentenyl diphosphate (IPP) in a ratio of 1:5 [15], as shown in Fig. 1. The pathway subsequently leads to the formation of many biomolecules including carotenoids, steroids, and ubiquinone. The enzyme 1-deoxy-d-xylulose-5-phosphate (DXP) synthase or DXS (EC 2.2.1.7) condenses glyceraldehyde-3-phosphate and pyruvate to synthesize DXP. This enzymatic reaction is the rate-limiting step and consequently, for an increased biosynthetic production rate of the end-products, a widespread interest has arisen in its research. Although the enzymes of the DXP pathway have been discovered two decades ago and have been studied in several microbes till now; however, their regulatory mechanism is still elusive. The DXS is the first enzyme of the DXP pathway belonging to the transferase family (EC 2.5.1.7). It catalyzes the condensation of G3P and pyruvate into the first intermediate of the pathway, i.e., DXP [15]. The DXP pathways enzymes, including DXS, are highly regulated. It has been suggested that the accumulation of DXS is regulated by other endogenous proteins, and perturbations of the growth conditions may affect its expression profile [16]. Besides playing a vital role in the biosynthesis of vitamin B1 and B6, it leads to the formation of isoprenoid precursors, and is thus functionally active at a crucial rate-limiting branch point of the pathway [17]. In contrast to an overwhelming orderly count of 2957 and 179,659 entries, existing in the Swissprot and TrEMBL databases of UniProtKB (May 14, 2020) [18, 19], only two DXS structures belonging to *Escherichia coli* (PDB ID: 2O1S) and *Deinococcus radiodurans* (PDB ID: 6OUW) could be determined through X-ray crystallography so far [20]. Besides sharing a high sequence identity of 45.47% and related catalytic activities, the two enzymes closely resemble each other (Fig. 2) [22]. To date, a wide range of isoprenoids have been produced from engineered *B. subtilis* for nutraceutical applications. However, the titers achieved till date from *B. subtilis* are far less than the ones achieved from *E. coli*, and it poses a major difficulty for their industrial scale up. The low yield of isoprenoids from engineered *B. subtilis* is a bottleneck for its industrial application [23]. It has been observed that DXS is a rate-limiting enzyme of DXP pathway because of its (i) low solubility; (ii) inhibition by IPP and DMAPP; and (iii) low turnover number [22]. The gene At3g47450, homologous to YqeH gene of *B. subtilis*, has been shown to regulate the accumulation of DXS in *Arabidopsis thaliana* [16]. Further, a negative correlation has been established between the Clp protease and DXP pathway enzymes. The Clp protease is also involved in the sporulation phase of *B. subtilis* [24], in which the biological production of isoprene decreases drastically [25]. This shows high endogenous regulation of DXP pathway enzymes, including DXS in *B. subtilis*.

The DXS enzyme is highly conserved, and the two proteins 621-residue 2O1S and 650-residue 6OUW share a topological similarity of 0.820 for 468 residues, as represented red and blue in Fig. 2, and their topological variation is localized across the loop regions and terminal overhangs. However, the DXS enzyme of *Bacillus subtilis* and its homologs are still not significantly explored for improving the catalytic activity. Although the three domains encoded by DXS share homology with the E1 subunit of pyruvate dehydrogenase and equivalent domains of transketolase (EC 2.2.1.1), their orientation is substantially different. The active site of DXS is present at the interface of domain I and II, unlike transketolase where it is present in the dimer interface [20]. Moreover, as recently shown, the active site of DXS is nearly twice the volume of transketolase and pyruvate dehydrogenase (EC 1.2.4.1) active sites [26, 27], and it should allow the biosynthesis of bulky molecules, making it an interesting target for the directed evolution methodologies. Hence, a careful scrutiny and dedicated dataset of homologs are urgently needed to accurately extract the closest entries to drive such methodologies.

To overcome the limitations and achieve an industrially high-yield (systematic) of isoprenoids from *B. subtilis*, there is an urgent need to computationally engineer its DXS enzyme. In this regard, the preliminary step is to screen its evolutionarily closest homologs for selecting the potent protein sequence(s) from its closest clade and tracing the highly conserved/variable sites so that a catalytically improved enzyme sequence could be redesigned through specific mutations proximal to its active site [28–31]*.* Due to the overabundant number of bacterial sequences other than *B. subtilis*, screening its functionally as well as topologically closest sequences is still a major challenge. A strategic comparative scrutiny thus becomes mandatory to screen the functionally closest set of natural variants for *Bacillus subtilis*.

In correlation with the cladistic divergence of the most predominant bacterial species *Enterobacteriaceae*, a non-redundant sequence dataset for *Bacillus subtilis* is constructed. The study focuses on the evolutionary diversification of the dataset and estimates the average branch length and topological conservation for all the resultant clades. It estimates the sequence conservation for the constructed dataset, and maps the active site residues within the well-studied representative DXS structure 6OUW. The co-evolving and hotspot residues are subsequently localized to analyze their degree of conservation and the key residues are designated for mutagenesis. Although it is impossible to explore the theoretical sequence space of a protein, the study will help to develop automated algorithms for decoding the functionally crucial sites of a protein. It would aid the construction of focused mutant library which will have a significant impact on generating catalytically improved enzymes in short time frames. Thus, the study is certainly the need of the hour for computationally evolving a catalytically improved DXS enzyme sequence with enhanced substrate affinity.

## Materials and methods

### Building the sequence dataset and alignment

**Fig. 3:**
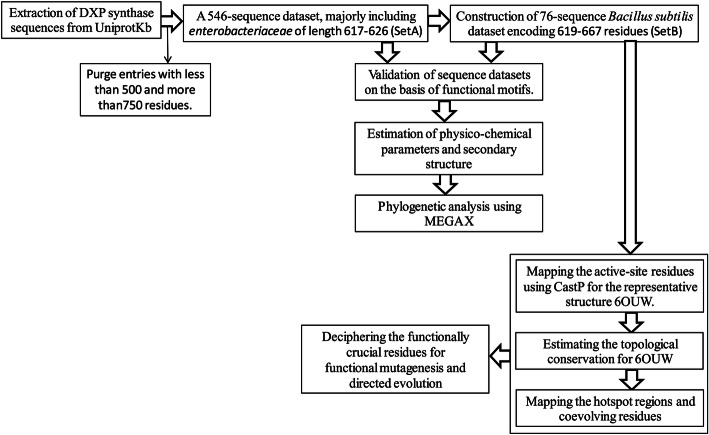
Algorithmic flowchart for the analysis from sequence dataset to functional annotation and coevolving residues across the active site

Screening the DXS protein in UniprotKB [32], a sequence dataset is constructed, as shown in the overall algorithmic flowchart of the study (Fig. 3). As per the sequence length of functionally similar protein structures, a robust length filter of 500-750 is deployed to purge all the functionally variant entries and build the dataset of 546 sequences (set A). It comprises 12, 11, 2, 2, 1, and 518 sequences from plant, animal, protista, human, fungi, and bacteria respectively, as enlisted in the Supplementary Table 1.

For all enzyme design protocols, the first and foremost challenge is to designate the evolutionarily conserved/variant regions and decipher the functionally important residues. For computationally evolving the proteins, it thus suggests the urgent need of building the evolutionarily closest sequence cluster and assesses the variation of sequences across the *Bacillus subtilis* in correlation with the most abundant set of proteins. As the bacterial sequences majorly predominate set A, belonging to the subfamily *Enterobacteriaceae*, it is considered to evolutionarily track the sequence divergence more effectively. Further, as this dataset is found to encode 617-626 residues, and the smaller sequence length is evolutionarily preferred [33], the entries are clustered as per their lengths to define two subsets set A_small_ and set A_large_. The sequences Q7VRH9.1 and Q8D357.1 are orderly selected as the representative set A entries for these subsets.

The 546-set sequence dataset is aligned using ClustalO server by deploying the default parameters (https://www.ebi.ac.uk/Tools/msa/clustalo/) [34, 35]. The server aligns the sequence dataset to derive the distances between the aligned residues and constructs a guide tree for further improving the alignment. The resultant sequence profile should thus yield a more reliable evolutionary relationship between the 546 sequences. To extrapolate the methodology to *Bacillus subtilis*, a set of 301 sequences are likewise retrieved and the redundant hits are purged through MMSeqs2 protocol [36]. It deploys a three-step cascaded workflow for mutually aligning the input sequences on the basis of an ungapped alignment and sensitive k-mer matching algorithm to yield the entries scoring higher than a given threshold. Purging the outliers, the final 76 sequence dataset (set B) is constructed. The entries are clustered as per their lengths and their sequence motifs are evaluated through the multiple EM for motif elicitation (MEME) server [37]. To confirm the functional annotation of the sequence dataset before deploying it for subsequent studies, the top three motifs of length 6-50 are screened by this online software suite. As the presence of signature sequence aids an initial computational verification of the function, the server increases the functional credibility of the sequence dataset. It yields 2 subsets, set B_small_ and set B_large_, orderly having a sequence length of 619 and 667 respectively, for the subsequent analysis, and the sequences AJW87412.1 and WP_007410329.1 are orderly selected as the representative entries.

### Sequence and structural analysis

Several physical and chemical parameters, viz., molecular weight, amino acid composition, extinction coefficient, estimated theoretical pI, and grade average of hydropathicity, aliphatic index, and instability index are important to estimate the physiochemical properties and topological features of a protein sequence. For the representative sequences of both datasets, the features are estimated through ProtParam (https://web.expasy.org/ProtParam) [38].

PSIPRED [39] is deployed to predict the three-state secondary structure for the selected representative sequences. It provides information corresponding to α-helices, β-sheets, coils, transmembrane helices, signal peptides, membrane interactions, re-entrant helix, and putative domain boundaries. For the representative sequences, the TMHMM server (http://www.cbs.dtu.dk/services/TMHMM/), based on the transmembrane hidden Markov model, is subsequently used to predict the integral transmembrane helices and discriminate between the soluble and membrane proteins [40]. Besides estimating the number of transmembrane helices, it predicts the expected number of transmembrane helix residues for the selected protein sequence [38].

### Evolutionary analysis

A phylogenetic tree is an estimate of the relationships among taxa/sequences and their hypothetical common ancestors. Most molecular phylogenetic trees estimate the statistically significant relationships among the species/sequences [41]. Molecular evolutionary genetics analysis (MEGA) is one such widely deployed tool to measure evolutionary distance among the sequences [42]. MEGA X is used to construct the interactive evolutionary trees for the datasets A and B. The constructed trees are visualized using the interactive tree of life (iTOL) server (https://itol.embl.de/) [43]. It is used to analyze the evolutionary relationships across the sequence datasets and distinctly highlight the species.

### Crucial residues for functional mutagenesis and directed evolution

DXS is an essential enzyme of the pathway and its expression is very rigidly controlled by the bacteria. Making its active site open to a variety of substrates will thus be phenomenal in increasing its productivity. To study the key substrate-binding residues, the experimentally solved structure, closest to the constructed sequence dataset of the functionally similar homologs of *Bacillus subtilis*, is screened from the PDB database through HHPred [44]. To functionally characterize and confirm the derived dataset, a MEME server is used to search and identify the previously unidentified motifs in the sequence dataset [37]. The motif length of 6-50 is used to localize the top three motifs.

The retrieved structure is subsequently fed to CASTp [45] for mapping the active site residues, lining the cavity. For localizing the conserved residues and their degree of conservation across the active site, Consurf [46] is deployed, and the average pairwise distance among the sequences, along with its lower and upper level, is estimated. For estimating the relative degree of sequence conservation, the experiment is repeated by including the 13 transketolase homologs. Functional divergence within and among these datasets is estimated as the level of evolutionary distant entries that usually emerge in such analysis. The functionally conserved and co-evolving residues are subsequently localized through the MISTIC approach using the mutual co-evolving information (MI) of the sequence profile [47]. MI is calculated between the residue columns and it reflects the extent of the co-evolutionary impact of one residue at another position within the MSA. Every node in the resultant network indicates a protein and their linker edge signifies the statistically significant similarity between them.

### Hotspot regions

A most crucial step of semi-rational directed evolution strategy is the selection of hotspot loci whose mutations lead to a significant improvement of the catalytic/biological activity of the proteins [48]. Hotspots are the sites where alanine mutations lead to an increase of at least 2 kcal/mol in the binding free energy. To analyze the mutational landscape and localize the hotspot regions within/proximal to the active site for the representative structure 6OUW, the hotspot server [49] is deployed for studying the functionally crucial and correlated hotspots. It will provide us a vital dataset to build the dedicated mutant libraries for the semi-rational directed evolution of these functionally similar enzymes.

To theoretically investigate the role of the identified hotspot residues on the biological stability of the representative structure 6OUW, their top-ranked missense mutations are deciphered through Popmusic (http://dezyme.com/en/software) [50]. On the basis of a linear combination of environment-specific statistical potentials and solvent accessibility of the mutated residue, it introduces a point mutation in the structure and estimates the resultant change in the thermodynamic stability in terms of ΔΔG score. The combinational assessment corrects its bias toward the destabilizing mutations, which usually impose physical symmetries under inverse mutations, and increases the robustness of the protocol [51, 52]. While the solvent accessibility is estimated within the range of 0 (buried) to 100 (*fully accessible*), the mutation effect on the protein stability is considered stabilizing if ΔΔG < 0. For strengthening the credibility of predictions, the Maestro server (https://pbwww.che.sbg.ac.at/maestro/web) is subsequently deployed [53]. On the basis of the statistical energy functions for the sequence and structural topology of the input protein, it estimates the difference in the folding free energy of the structure upon mutations along with a confidence score through multiple linear regression, SVM, and neural networks [54]. It scrutinizes the stabilizing disulfide bonds and free energy change through a high-throughput scanning of multi-point mutations [53]. Maestro predictions are less biased relative to the group of inverse mutations [55]. To the best of our knowledge, no tool suggests the coupled mutations for introducing the new stabilizing contacts within the structure.

## Results and discussion

### Sequence and structural analysis

The computational methods aid a swift characterization and estimation of the functional properties of protein sequences. The physicochemical properties, viz., pI value, extinction coefficient, molecular weight, average hydrophobicity, and instability index are estimated through the ProtParam server for the representative sequences of set A and set B to determine their uniqueness (Table 1), as has been recently shown [48]. Isoelectric point or pI is the pH where the protein molecule has no net charge. The pI value higher and lower than 7.0 orderly indicates the alkaline and acidic character of a protein respectively. While the theoretical pI of *Bacillus subtilis* sequences shows a pI of 5.80-5.91, indicating the acidic nature, the *Enterobacteria* sequences display a significantly higher basic pI of 9.34-9.57. It indicates a highly narrow range of sequence variation within the species, and a significantly diverse range of variations across various species. It has been reported that an instability index score of less than 40 confirms the structural stability of a protein [37]. The representatives for set A (Q7VRH9.1 and Q8D357.1) and set B (AJW87412.1 and WP_007410329.1) exhibit a score within the range of 30-40 (Table 1), indicating their cellular stability. An interrelation is observed between the stability and half-life, and the stable proteins manifesting an in vivo half-life of at least 16 h, are usually expected to express an aliphatic index of less than 40 [49]. The inherent stability of these proteins provides an added benefit of minimizing the experimental costs and steps, usually deployed for such studies.

**Table 1:** Physicochemical parameters, estimated by ProtParam, indicating a substantial variation for certain features

Sequence accession ID (set)	Amino acids	Molecular weight (KDa)	Extinction coefficient (M−^**1**^ cm−^**1**^)	Theoretical pI	GRAVY score	Aliphatic index	Instability index
**Q7VRH9.1 (set A)**	617	68.722	64220	9.34	−0.122	98.98	34.64
**Q8D357.1 (set A)**	626	69.851	56520	9.57	−0.054	103.74	34.09
**AJW87412.1 (set B)**	619	67.913	53985	5.99	−0.149	88.74	33.30
**WP_007410329.1 (set B)**	667	72.584	91790	5.13	−0.269	79.48	36.68

**Fig. 4:**
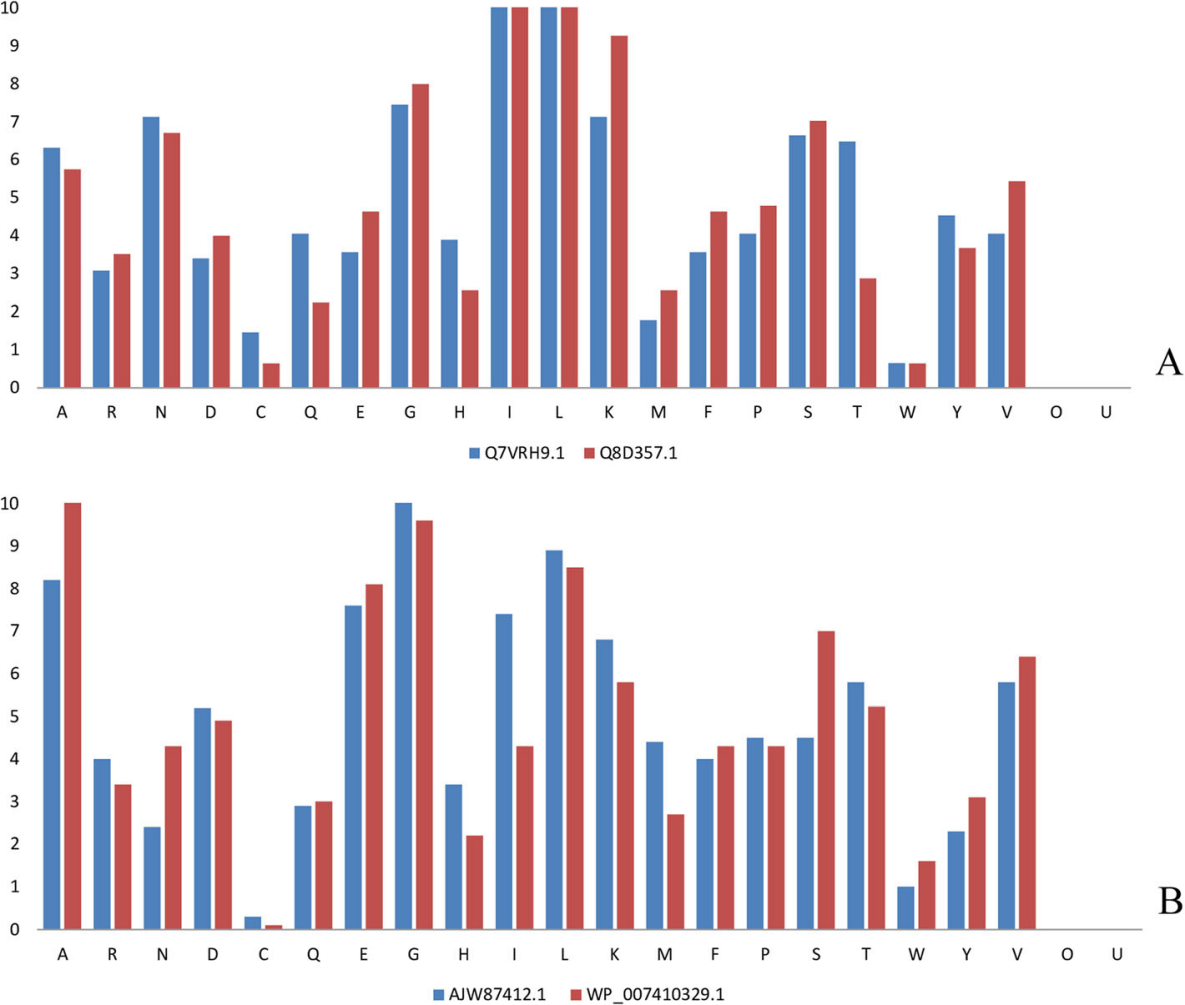
Variation of residue frequencies between the representative sequences Q7VRH9.1 and Q8D357.1 and AJW87412.1 and WP_007410329.1 of the two datasets, belonging to *Enterobacteriaceae* and *Bacillus* species respectively. A substantial variation in the residue percentages is observed for a few residues within and between the two datasets

The average extinction coefficient (measured as M^−1^ cm^−1^) indicates the quantity of light absorbed at 280 nm, and it is found to be significantly different for the representative set A and set B sequences (Table 1). The score is dependent on the frequency of cysteine, tryptophan, and tyrosine residues, and it aids a deeper quantitative understanding of the interactions against any other protein/ligand. The aliphatic index is subsequently estimated for these proteins and it indicates a constricted index range of 79.48-88.74 and 98.98-103.74 for the *Bacillus subtilis* and *Enterobacteriaceae* sequences respectively*.* The aliphatic index is the relative volume occupied by the aliphatic amino acids, and the estimations show a bit lower thermostability for the *Bacillus subtilis* sequences. Further, the set A representative sequences illustrate a diverse range of the grand average of hydrophobicity (GRAVY) scores from −0.054 to −0.122, in contrast to the respective range of −0.149 to −0.269 for the set B sequences. The negative GRAVY score signifies the nonpolar nature of a protein molecule. It indicates the energetically favorable interactions with the hydrophilic water molecules, and it thus shows that the hydrophobic residues are robustly conserved in *Bacillus subtilis* sequences [56]. Further, the residue composition analysis orderly shows a high proportion of glycine, 10.70% and 10.54% for set A (Fig. 4a) and 10.50% and 9.6% for set B (Fig. 4b). It indicates that the sequences encode a very low number of charged residues and hence, their extracellular solubility should be too low within the solvent.

**Fig. 5:**
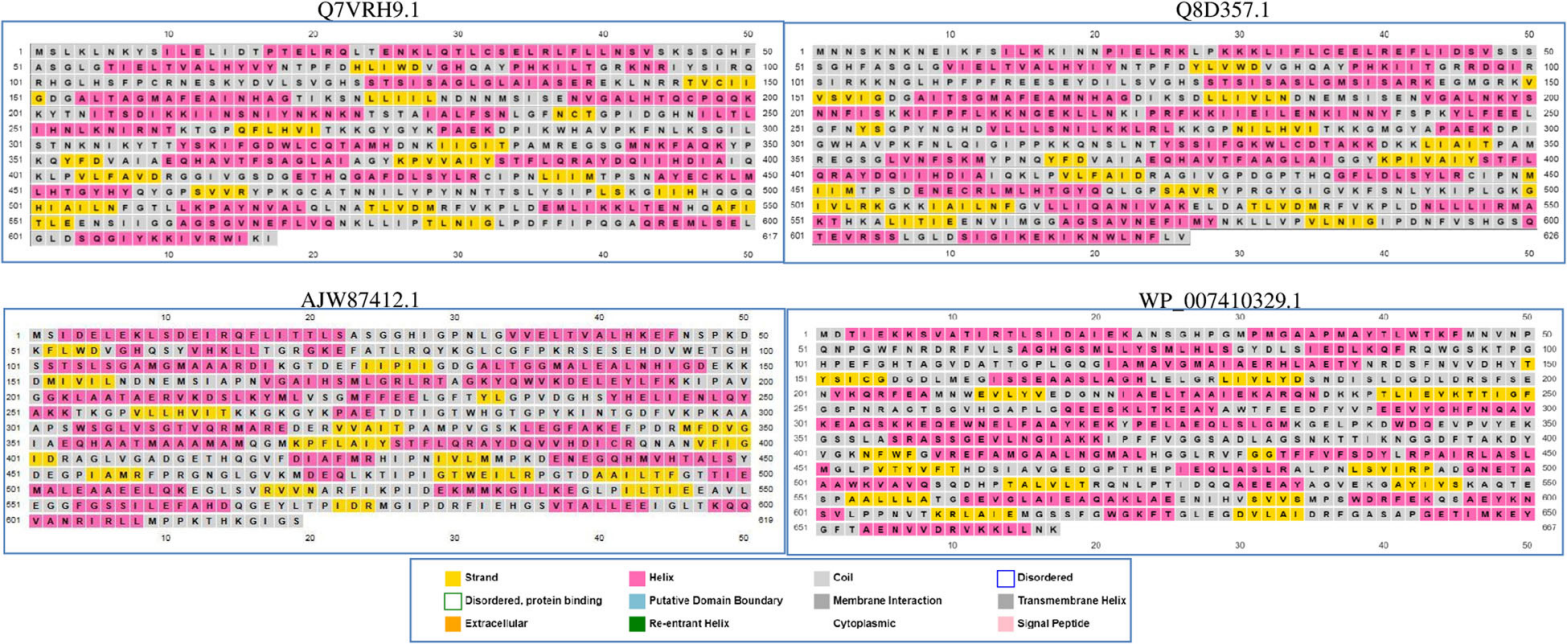
PSIPRED based estimation of the secondary structure for the representative sequences (**a**) Q7VRH9.1, (**b**) Q8D357.1, (**c**) AJW87412.1, and (**d**) WP_007410329.1 of the two sets A and B

**Fig. 6:**
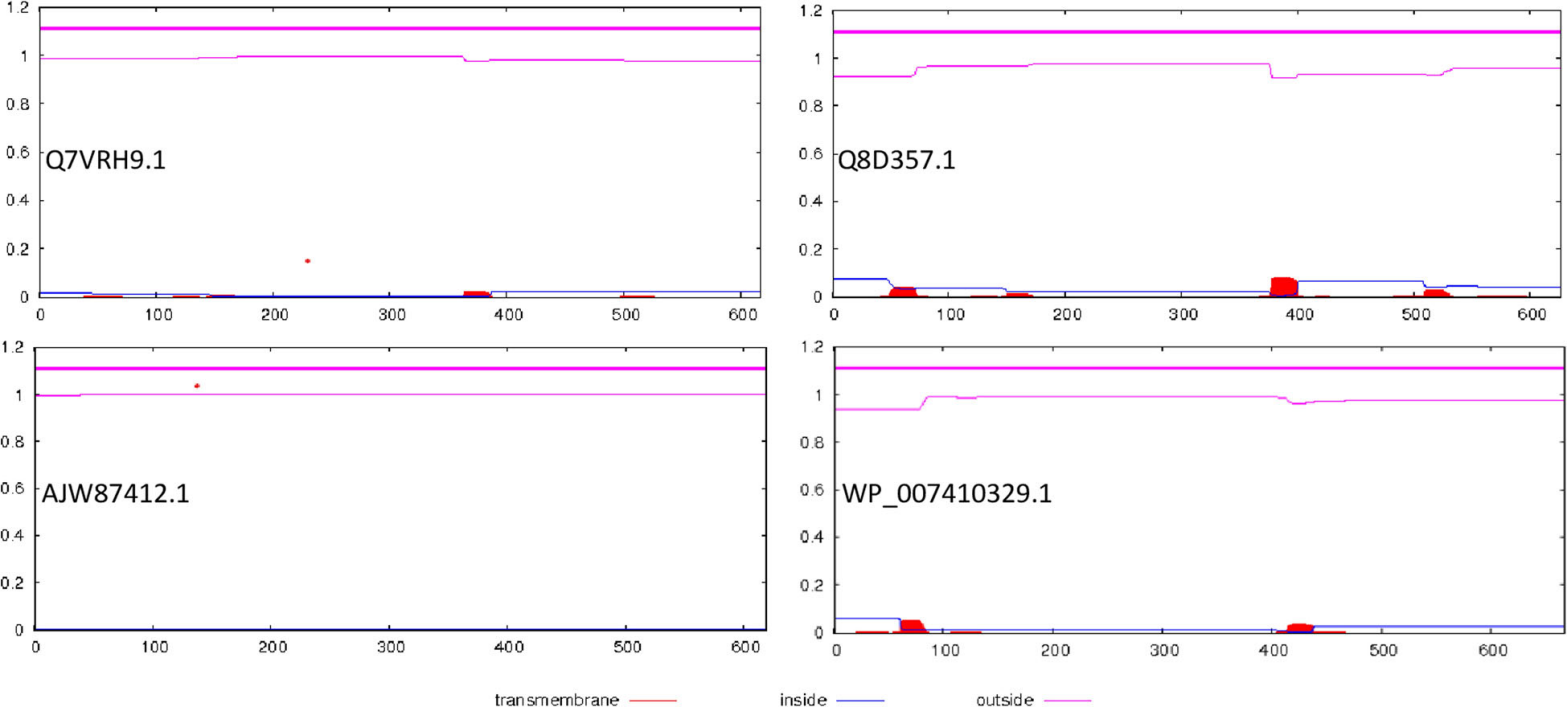
Prediction of protein transmembrane structure for representative sequences Q7VRH9.1, and Q8D357.1, and AJW87412.1 and WP_007410329.1

PSIPRED is further used to predict the secondary structure of the representative sequences of the two datasets (Fig. 5). The secondary structure elements, viz., helix, sheet, and coil define a protein structure and play a key role in the design of various bioanalytical experiments. A residue fraction of 42.78% and 13.12% orderly defines the helical and stranded substructure of Q7VRH9.1, in contrast to the respective proportion of 43.61% and 12.93% for Q8D357.1. For set B also, a fraction of 43.29% helix and 12.76% strand residues are encoded in AJW87412.1 in comparison to the respective proportion of 43.62% and 11.69% for WP_007410329.1. It suggests a substantial predominance of the helical residues within this class of proteins. The transmembrane helical regions are further localized for the representative sequences through TMHMM, and sequences Q7VRH9.1 and Q8D357.1 are found to respectively encode a fraction of 0.863 and 3.647 transmembrane helical residues (Fig. 6), in contrast to the respective proportion of 0.13269 and 2.12531 of the set B sequences AJW87412.1 and WP_007410329.1.

### Evolutionary analysis

**Fig. 7:**
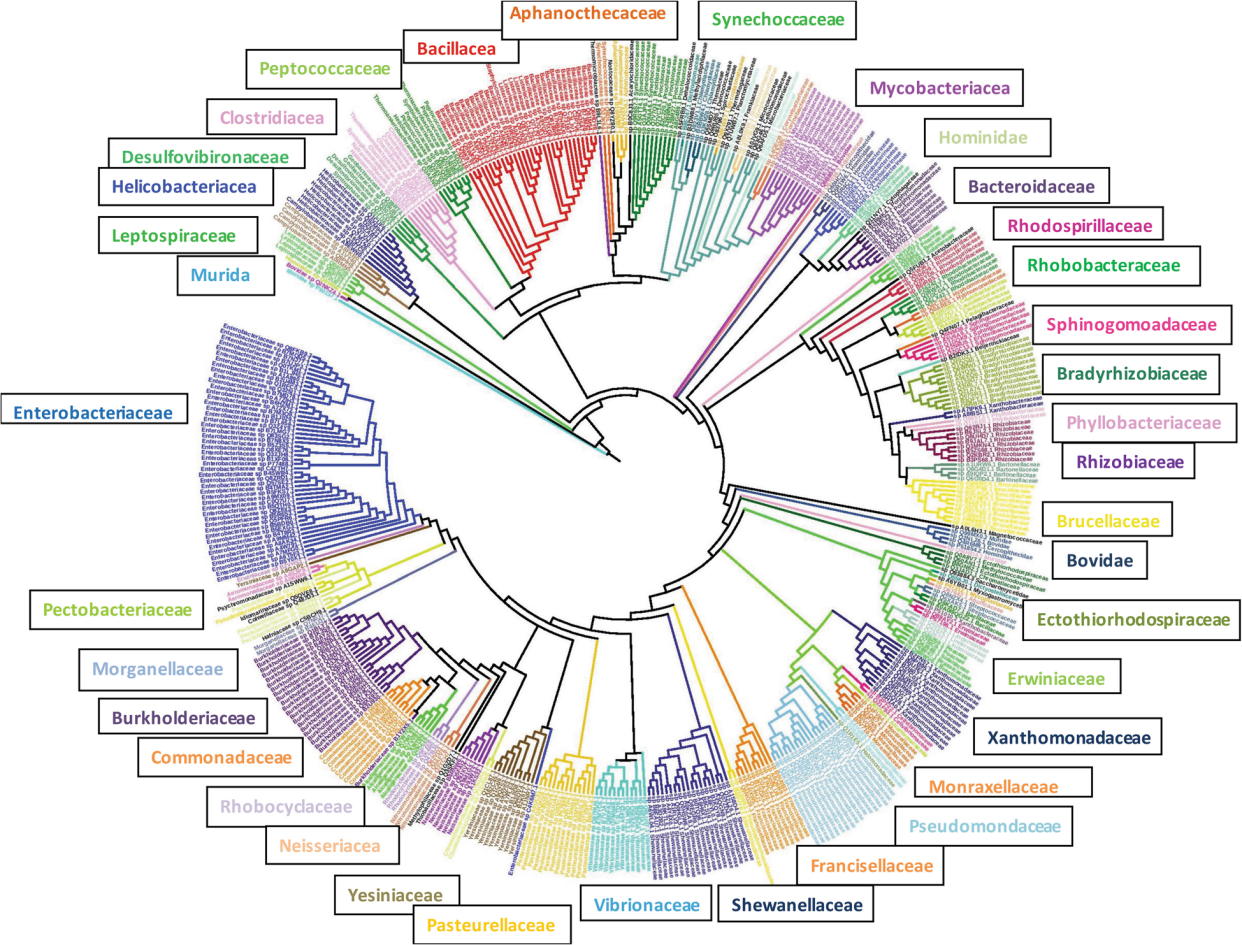
Phylogenetic tree of the 546 sequence dataset of DXP synthase. An average pairwise score of 1.012 within the range of 1.0175 to 2.876 is found

**Fig. 8:**
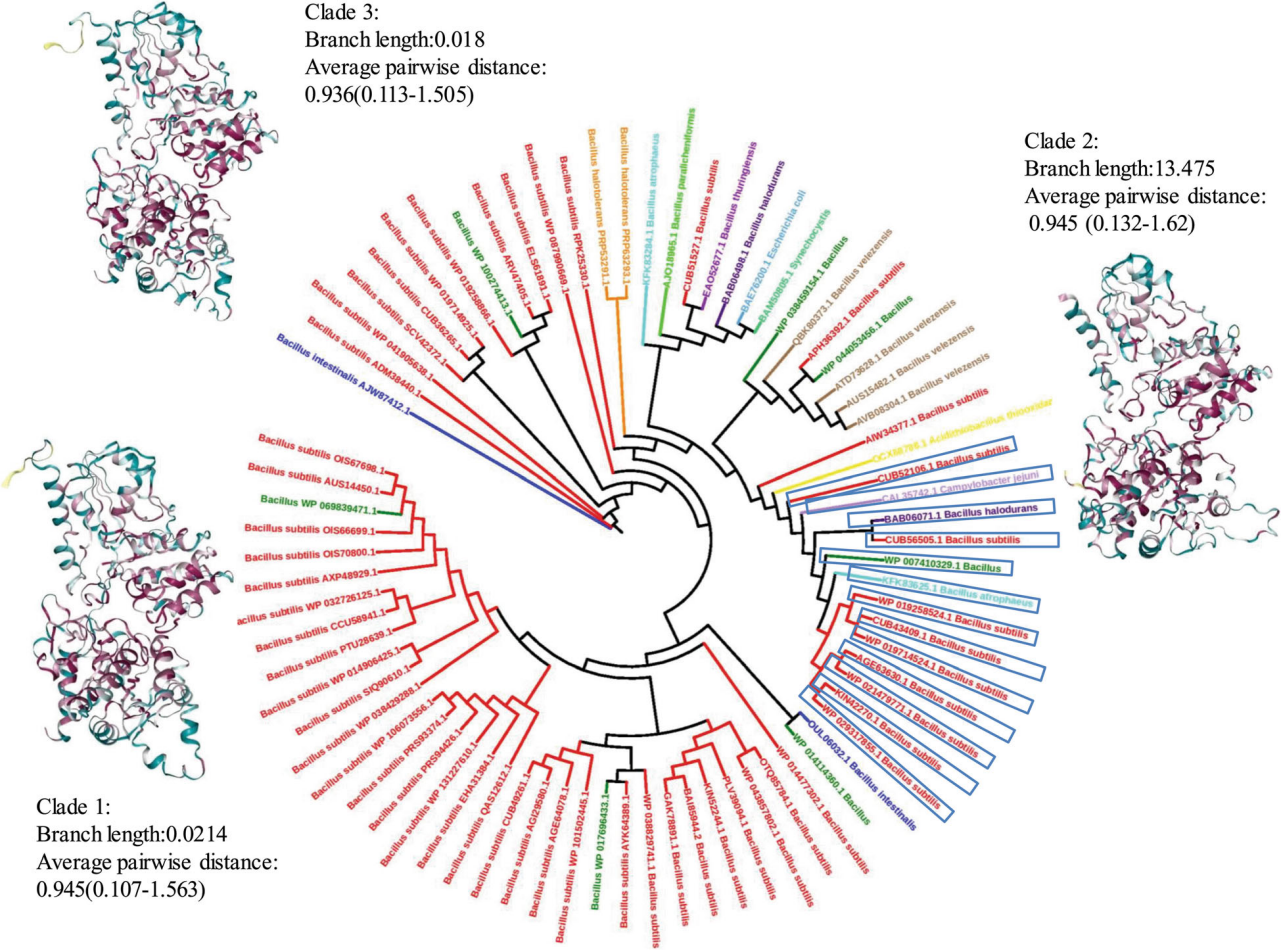
Phylogenetic tree of 76 sequences of DXP synthase proteins in *Bacillus subtilis.* The 13 transketolase sequences are marked with blue squared boxes, and it shows a clear segregation of the two enzymes

Screening the DXS protein in UniprotKB [32], it results in 551 sequences with sequence length ranging from 519 to 741. The sequence length range of the dataset varies from 106-684 and a large majority (84.615%) is found to be within 600-700. Purging the functional outliers, it yields 546 sequence dataset whose alignment is fed to MEGA X to derive the evolutionary relationship. Although computing an evolutionarily optimal tree topology is termed to be an NP-hard combinatorial optimization problem [57], the maximum likelihood method [58] has been well proven to yield the robustly accurate results for the sequence dataset than the other methods [59, 60] and is therefore deployed to infer the phylogenetic history of the dataset. The JTT substitution matrix [61] is utilized for estimating the evolutionary distances within the dataset at 4 discrete gamma distribution categories for the residue substitution. Bootstrap resampling (1000 replicates) [62] is used to assess the robustness of the groupings. It integrates the replicate trees for the clustered set of associated taxa into the bootstrap test to show its percentage next to the branches. The grouped set of sequences is found to encode a substantial sequence similarity in comparison to the other dataset entries and is expected to share a statistically significant evolutionary relationship. The tree is subsequently visualized in the IToL server for analyzing it further (Fig. 7). As nature tends to decrease the sequence lengths of proteins for saving the energy required to synthesize and fold longer proteins [63], the shortest sequences of various clades should be prioritized to evaluate their evolutionary relationships. For the inferred 37 clades, three shortest sequences are further selected to build a new dataset of 108 sequences, and their functional similarity is confirmed through their motifs to further evaluate their evolutionary relationships. The resultant tree shows significantly distant associations across 37 clades belonging to *Leptospiraceae*, *Helicobacteriacea*, *Desulfovibrionaceae*, *Clostridiacea*, *Peptococccacea*, *Bacillacea*, *Aphanocthecaceae*, *Synechoccaceae*, *Mycobacteriaceae*, *Hominidae*, *Bacteroidaceae*, *Rhodospirillaceae*, *Rhodobacteraceae*, *Sphinogomoadace*, *Bradyrhizobiaceae*, *Phyllobacteriaceae*, *Rhizobiaceae*, *Brucellaceae*, *Bovidae*, *Ectothiorhodospiraceae*, *Erwiniaceae*, *Xanthomonadaceae*, *Monraxellaceae*, *Pseudomondaceae*, *Francisellaceae*, *Shewanellaceae*, *Vibrionaceae*, *Pasteurellaceae*, *Yesiniaceae*, *Neisseriacea*, *Rhobocyclaceae*, *Commonadaceae*, *Burkholderiacea*, *Morganellaceae*, *Pectobacteriaceae*, *Enterobacteriaceae*, and *Murida* species. The log-likelihood and total tree length scores for the tree are found to be −56151.89 and 33.750 respectively. Similarly, the tree is constructed for the 76 sequence dataset (set B) and visualized in the IToL server. Set B is found to evolve in three distinct clades. The log-likelihood score and total tree length of its consensus tree solution are found to be −15262.705 and 20.657 respectively. As per the evolutionary trees of set A and set B, it should be logical to state that these datasets are divergent, and extending the sequence space to set A may prove to be incorrect. As speculated, the strategy has worked well, and despite sharing significant homology with DXS [64], a set of well-clustered sets of only 13 transketolase is found, as marked with blue squared boxes in Fig. 8. It clearly indicates clear segregation of these sequences from the other entries, and further prioritization of the closest sequence space of DXS [65] is thus possible from the derived alternative residue dataset. The derived dataset further indicate a sequence identity within the range of 42.16-99.84 against 6OUW, and again proves an evolutionarily significant closeness of data. Our results thus show a significant evolutionary diversification of DXS across various species, in contrast to *Bacillus subtilis*, and this is crucial to derive a detailed dataset of the encoded alternative residues for constructing the mutant library.

### Crucial residues for functional mutagenesis and directed evolution

**Fig. 9:**
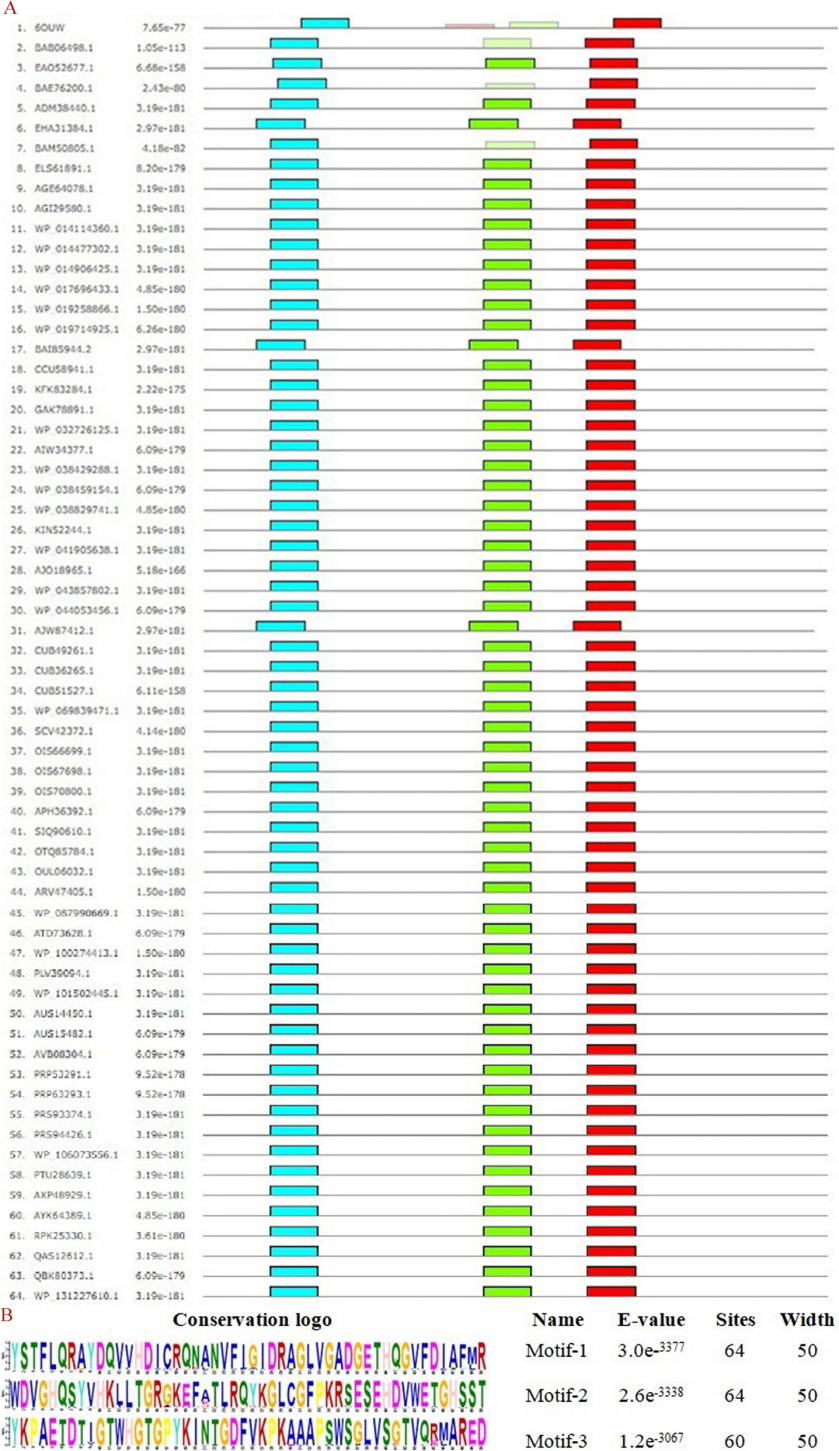
**a** Statistically significant occurrence of tandem motifs within the 63 DXS sequences of *Bacillus subtilis,* in correlation with the reference structure 6OUW. Motifs 1 and 3 are found statistically conserved across all homologs. **b** Conservation logo and statistical *E* value scores of localized motifs

DXS is a very crucial rate-limiting enzyme of the methylerythritol phosphate (MEP) pathway and its expression is very rigidly controlled by the bacteria. Rather than modulating the active site, its overexpression has been widely deployed to increase the bacterial production of carotenoids [66, 67]. To reliably analyze the active site for mutagenesis, the experimentally solved structure closest to the profile of the 63 sequence dataset of *B. subtilis* is screened through HHPred. With an *E* value and sequence identity of 7.3e−90 and 42% respectively, the template having PDB ID: 6OUW is found to be the closest protein structure [68]. For confirming the functional similarity of the constructed 63 sequence dataset of *B. subtilis*, the top three motifs are identified through MEME suite (Fig. 9a). The three potential motifs are designated as 1, 2, and 3 (Fig. 9b), and motif2 is not found to demonstrate a statistically significant conservation score in 4 sequences, including the reference protein structure. Within the reference structure, motifs 1 and 3 are orderly located between residues 99-148 and 416-465, and appear to play a functionally significant role in domain1 and domain2 respectively [20]. Against the reference structure 6OUW, the constructed sequence alignment is fed to ESPRIPT3 [69] and it presents the conservation of only a few residues, scattered across the chain (Supplementary Fig. S1).

**Fig. 10:**
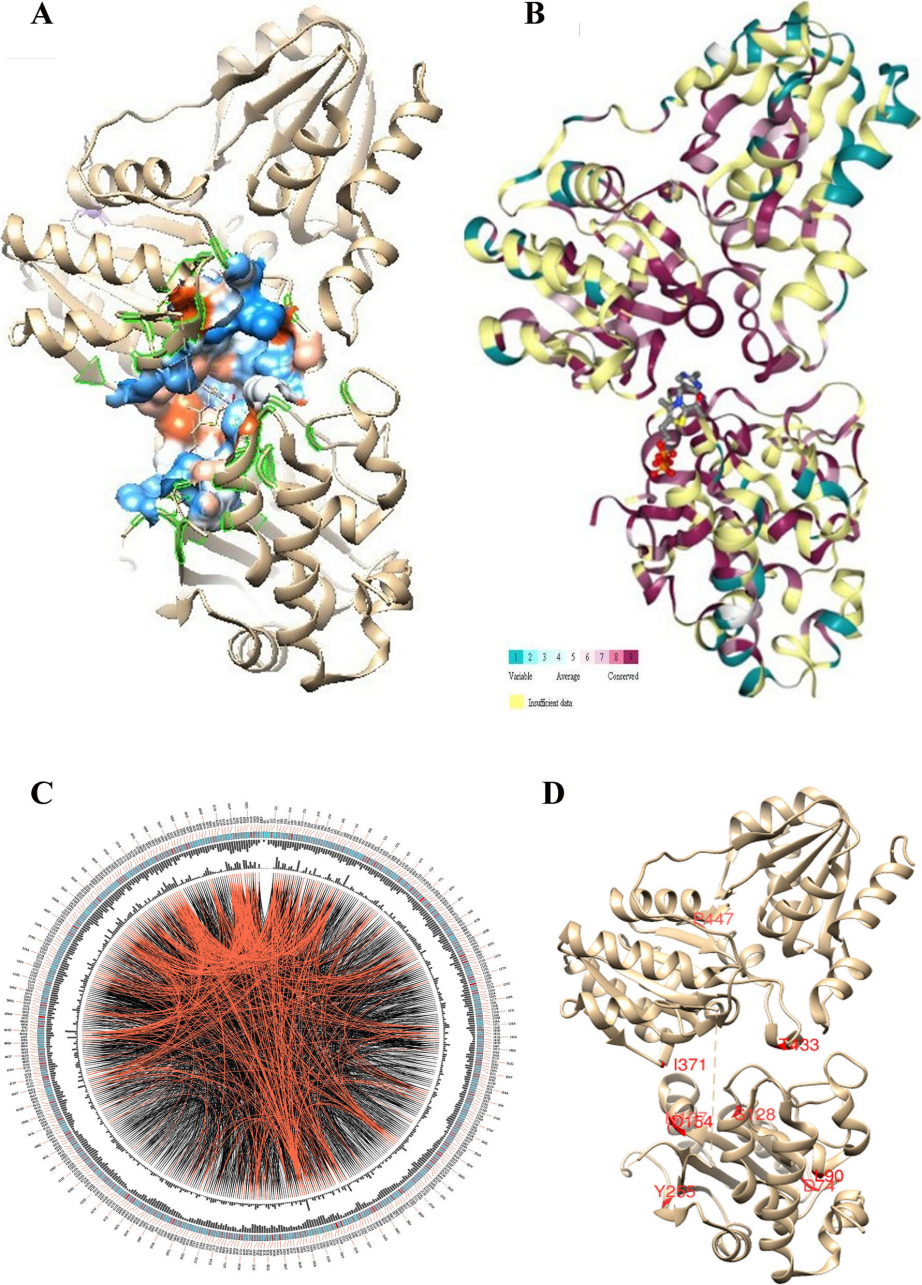
Schematic representation of the active site residues of the representative structure 6OUW. **a** Active site protein pocket yielded by CastP [45]. **b** Conservation profile of Consurf [46], maroon being the most conserved residue. **c** MI network [47] of the conserved coevolved residues encoded in 63 DXS sequences. **a** Labels outward of the second circle represent the residue loci, and the colored square boxes indicate the level of sequence conservation within the intensity range of low (blue) to high (red). The two internal circles show proximity mutual information and cumulative mutual information scores respectively. As per MISTIC protocol, the curved central linkers connect residues with statistically significant MI scores (> 6.5), with red, black, and gray orderly indicating the residue pairs with the top 5% scores, average scores between 70 and 95% and lowest scores. **d** Structural localization of the 10 conserved coevolving residues, indicating the most preserved for directed evolution methodologies

To decode the vital structural residues across the sequence profile, CASTp [45] server is used to localize the active site of the representative structure 6OUW (Fig. 10a). The top-ranked resultant pocket shows a surface area and volume of 1487.0 Å^2^ and 3057.5 Å^3^ respectively. A set of 61 residues, viz., GLY48, GLY49, LEU50, HIS51, LEU52, ALA53, SER54, GLY57, ASP79, VAL80, HIS82, GLN83, LYS101, PHE109, GLY123, HIS124, ALA125, VAL151, GLY153, ASP154, GLY155, SER156, ASN181, ASN183, GLU184, THR287, LYS289, ASP310, THR313, E315, TYR316, VAL317, PRO318, ALA321, SER323, TRP324, SER325, PRP347, ALA348, MET349, ARG350, GLN351, GLY352, ASP368, ILE371, GLU373, ILE394, TYR395, PHE398, ARG401, ASP422, ARG423, VAL427, ALA429, ASP430, HIS434, PRO479, ARG480, GLY481, and ASN482 are found lining the active site in between the domain1 and domain2 regions. While the domain1 is a parallel β-sheet structure of five strands ranging from residues 1-319, the domain2 is a six-stranded parallel β-sheet structure defined by 176 residues (320-495) [20]. To decipher the most crucial mutations proximal to this site, the structural conservation is estimated for the 63 sequence dataset, by considering and excluding the transketolase sequences against 6OUW through Consurf [46]. While the former shows an average pairwise distance of 0.701 among the sequences, with a lower and upper bound of 1.017e−07 and 2.442 respectively, the latter set shows an average distance of 0.137 within the range of 1.017e−07-1.040. It confirms the evolutionarily significant closeness of our constructed dataset, and the transketolase structures are hereby shown to have a substantial evolutionary divergence from DXS sequences. Further, 114 positions LEU30, ARG38, HIS51, LEU56, VAL59, ALA64, LEU65, ASP74, ASP79, VAL80, HIS82, GLN83, TYR85, HIS87, LYS88, LEU90, THR91, ARG93, GLU114, SER115, ASP118, HIS124, SER126, THR127, SER128, ALA136, ALA138, ILE152, ASP154, THR158, MET161, ALA162, ALA165, ASN167, LEU180-NDNEMS-ILE187, ASN190, VAL191, ALA193, TYR255, ASP260, HIS262, LEU267, PRO280, HIS284, THR287, LYS289, ALA296, GLU297, ASP299, HIS304, SER325, ALA336, THR346, ALA348, MET349, PRO363, ASP368, VAL369, ILE371, ALA372, GLU373, ALA376, THR378, ALA380, ALA384, PRO390, ALA393, TYR395, SER396, THR397, PHE398, LEU399, GLN400, ARG401, TYR403, ASP404, GLN405, HIS408, ASP409, VAL417, ASP422, ARG423, VAL427, ALA429, ASP430, THR433, HIS434, ASP439, ARG444, PRO447, PRO454, ASP456, GLU459, ALA475, ARG477, ARG480, ASN482, LEU527, ARG536, LYS539, PRO540, ASP542, THR558, GLU560, GLU574, PRO591, and ASP592 are found to be completely conserved, 58 constituting domain1 and 46 within domain2, as shown maroon in the color grade panel of Fig. 10b.

Screening the residues interacting with its natively bound ligand 2-acetyl-thiamine diphosphate through the protein-ligand interaction profiler server [70], it shows a salt-bridge, π-stacking, and hydrophobic bond of 4.66 Å and 3.61 Å, 3.59 Å and 3.51 Å for HIS82 and LYS289, PHE398 and VAL80 residues respectively. Moreover, this ligand is found interacting with SER54, GLY123, ALA125, GLY153, GLY155, SER156, and ASN183 residues through hydrogen bond within a distance range of 1.76-3.57 Å. Although Consurf indicates a complete conservation for all these residues, only VAL80, HIS82, LYS289, and PHE398 are found lining the active site by CastP, making an interaction fingerprint. It indicates that our study correctly extracts all the functionally crucial residues across the active site.

The active site of this enzyme is shown to be highly conserved [71], and as shown by CastP, the 61-residue set majorly defines the active site cavity along with the less conserved proximal residues to make it voluminous. It thus becomes reasonable to state that several sequence loci evolutionarily experience a small degree of sequence modifications, and to further substantiate the functionally significant co-evolving residues across the constructed sequence dataset, MISTIC algorithm is used [47]. To discriminate the functionally significant co-evolving residues and the ones having the phylogenetic linkage, the resultant statistical scores are adjusted through the average product correction (APC) [72, 73]. The resultant residue network (Fig. 10c) indicates connections among several residues, and it may emerge due to factors including the phylogenetically preferred substitutions and topological stabilization constraints. Excavating it further, 10 positions ASP74, LEU90, SER128, ASP154, ASN167, ASN190, TYR255, ILE371, THR433, and PRO447 are found to be completely conserved (Fig. 10d). Moreover, only ASP154 and ILE371 are found to be the conserved coevolving residues within the active site. DXS is shown to be highly specific for its substrates [74] and this study will pave way to increase the promiscuity of its active site. The study will certainly be useful to gather data regarding the functionally crucial residues for several other functional studies [75–81]. It would also allow in extracting the evolutionarily closest templates for the protein sequences and would aid to improve the accuracy of the conventional template-based protein modeling protocols [82–90]. The strategy would be significantly useful for improving the algorithmic accuracy of several related research works including protein modeling [91] and folding [92] and functional enzymatic characterization [93–100].

### Functional hotspots

Hotspot server [34] is used to localize the hotspot regions within the representative structure 6OUW. Excluding the buried and the correlated loci, THR7, SER8, ASP9, ARG47, THR288, LYS291, PRO318, GLU596, VAL625, and PRO626 appear to be the most flexible residues, and none of these positions are found within the catalytic pocket. Among the 114 completely conserved loci, a correlated hotspot is observed between the two residues, ALA376 and THR378 through an unconserved residue VAL375 to make an interlinked evolutionarily correlated triad, and ASP422 and ARG423 are also found to form a correlated set. Although away from the active site, these residues are likely to have an impact on the catalytic activity, and this needs to be experimentally validated for getting further insights.

The PopMuSiC3.1 and MAESTRO servers are used to assess the functional impact of mutations on the representative structure 6OUW. At the first stage, the top-ranked mutations for the 14 identified hotspot residues are evaluated. Besides evaluating the ΔΔG score (Kcal/mol) for the mutations through Popmusic and MAESTRO protocols, the secondary structure and solvent accessibility and confidence score of the residue are orderly estimated through these two algorithms to predict the overall effect of a mutation. As shown by Popmusic, a total of 11 mutations are estimated to have a negative ΔΔG score (*negative score correspond to increased thermodynamic stability*). However, MAESTRO indicates a complete agreement for only 5 of these variations. A value lower than zero indicates the stabilizing mutation and only mutations 7-11 are found to be thermodynamically stable (Table 2). Further, Popmusic is recently proven to be a reliable and robust algorithm [101], and hence strategically deploying the scores on the basis of its scores would be more accurate.

**Table 2:** List of predicted top-ranked mutations for the 14 hotspot residues

#	Residues	Top-ranked mutations and scores		Crucial sequence/structural property
		PopMuSiC3.1	MAESTRO	
		ΔΔG (Kcal/mol); secondary structure, solvent accessibility (%)	ΔΔG (Kcal/mol), confidence score	
1	THR7	T7C = −0.11; C, 100	0.718, 0.731	Highly flexible
2	SER8	S8C = 0.07; C, 42.11	0.473, 0.837	Highly flexible
3	ASP9	D9P = −0.1; S, 87.93	0.352, 0.776	Highly flexible
4	ARG47	R47L = −0.76; S, 42.85	0.552, 0.808	Completely conserved and highly flexible
5	THR288	T288C = 0.06; C, 53.6	−0.144, 0.933	Highly flexible
6	LYS291	K291R = −0.1; C, 51.9	0.041, 0.880	Highly flexible
7	PRO318	P318D = −0.16; C, 59.37	−0.115, 0.923	Completely conserved, highly flexible
8	ALA376	A376Y = −0.33; H, 0	−0.489, 0.863	Completely conserved and forms a correlated hotspot with THR378
9	THR378	T378Q = −0.13; H, 34.15	−0.615, 0.868	Completely conserved and forms a correlated hotspot with ALA376
10	ASP422	D422C = −1.28; E, 0	−0.667, 0.880	Completely conserved and forms a correlated hotspot with ARG423
11	ARG423	R423Y = −0.26; S, 18.17	−0.190, 0.904	Completely conserved and forms a correlated hotspot with ASP422
12	GLU596	E596P = −0.49; C, 50.8	0.063, 0.890	Highly flexible
13	VAL625	V625I = 0.45; C, 20.73	−0.058, 0.932	Highly flexible
14	PRO626	P626T = −0.01; C, 96.03	0.200, 0.840	Highly flexible

Evaluating the 5 top-ranked mutations 7-11, it is observed that all these positions are completely conserved in the functionally similar sequence profile of the representative structure 6OUW. The mutation D422C shows a ΔΔG score of −1.28 and is predicted to be the most stabilizing mutation by Popmusic. MAESTRO also confirms this prediction and shows the lowest ΔΔG score of −0.667 with a high confidence score of 0.880. However, it is found to be a completely buried β-sheet residue within the active site, and its mutation may prove to be deleterious. Further, the conserved residues ALA376 and THR378 are found to form an evolutionarily correlated triad through an unconserved residue VAL375, and ASP422 and ARG423 are also found to be correlated loci. With an orderly solvent accessibility score of 0, 34.15, 0, and 18.17, these positions form the helix, helix, strand, and structural bend substructure. Hence, for their crucial role in building the overall conformation, these positions are predicted to be not the best ones for a mutagenesis in vitro. Lastly, MAESTRO shows the highest confidence score of 0.923 for P318D. Popmusic and MAESTRO algorithms show an orderly ΔΔG score of −0.16 and −0.115 for this mutation, and it indicates a satisfactory stabilizing effect. More importantly, although it is an active site position, it is localized over a coil segment with a significantly high solvent accessibility of 59.37. Hence, with the highest confidence score of 0.923, P318D should be the first ideal choice for performing a mutagenesis. Stabilizing this highly flexible coiled residue would thus impart a structural stability to 6OUW, as has been planned several times [102–104].

## Conclusion

The study performs a sequence and structure-based analysis of the DXS sequence of *Bacillus subtilis* in comparison to its most prevalent bacterial orthologues. The pipeline used for the study incorporates evolutionary analysis of *B. subtilis* sequences with the other usually encountered bacterial sequences and transketolase. Sequence and structural analysis indicate that only 5 of the 14 identified hotspot positions are completely conserved and 10 positions are highly flexible. Analysis of the top-ranked missense mutations for the 14 hotspots through POPMUSIC and MAESTRO affirm the biological credibility of only 5 mutations, of which, VAL375, ALA376, and THR378 form an evolutionarily correlated triad, and ASP422 and ARG423 are found to be correlated pair. PRO318 is present in the active site and is one of the most flexible residues. The P318D mutation indicates a higher chance of improving the thermostability of DXS. Since DXS is the most crucial enzyme to direct the carbon flux toward the biosynthesis of terpenoids in *B. subtilis*, the present study might be helpful to develop its functionally improved variants for improving the microbial production of terpenoid-based flavoring, fragrance, and therapeutic compounds.

## Supplementary Information

**Additional file 1: Supplementary Table 1.** List of sequences from plant, animal, protista, human, fungi, and bacteria

**Additional file 2: Figure S1.** Multiple sequence alignment of the constructed dataset against the reference structure 6OUW. The alignment is constructed through ClustalO algorithm and is parsed against 6OUW through Espript3 server. The red shading indicates the sequence conservation.

## Data Availability

The constructed files/datasets analyzed in this study are available from the corresponding author on reasonable request.

## Abbreviations

GRAS: Generally regarded as safe
DXS: 1-deoxy-D-xylulose-5-phosphate synthase
IUCN: International Union for Conservation of Nature
DXP: 1-deoxy-D-xylulose-5-phosphate
G3P: Glyceraldehyde-3-phosphate
DMAPP: Dimethylallyl diphosphate
IPP: Isopentenyl diphosphate
MEP: Methylerythritol phosphate
MEGA: Molecular evolutionary genetics analysis
ITOL: Interactive tree of life
MEME: Multiple EM for motif elicitation
GRAVY: Grand average of hydrophobicity
APC: Average product correction
