# Supplementary material for: Excavating the functionally crucial active-site residues of the DXS protein of Bacillus subtilis by exploring its closest homologues
Source: J Genet Eng Biotechnol. 2020 Nov 26;18:76. doi: 10.1186/s43141-020-00087-x (PMC7691408; doi:10.1186/s43141-020-00087-x)
Supplement: Supplementary file 1 — Additional file 1: Supplementary Table 1. List of sequences from plant, animal, protista, human, fungi, and bacteria [file 43141_2020_87_MOESM1_ESM.docx]

| **Species** | **Strength** |
| --- | --- |
| Brassicaceae | 3 |
| Deinococcaceae | 2 |
| Enterobacteriaceae | 51 |
| Chlamydiaceae | 9 |
| Thermotogaceae | 3 |
| Bacillaceae | 24 |
| Corynebacteriaceae | 5 |
| Synechococcaceae | 12 |
| Pasteurellaceae | 13 |
| Listeriaceae | 5 |
| Rhizobiaceae | 8 |
| Methylacidiphilaceae | 1 |
| Pseudomonadaceae | 17 |
| Burkholderiaceae | 24 |
| Bacillatesincertaesedis | 2 |
| Moraxellaceae | 5 |
| Magnetococcaceae | 1 |
| Pectobacteriaceae | 3 |
| Aphanothecaceae | 3 |
| Shewanellaceae | 19 |
| Micrococcaceae | 5 |
| Pseudoalteromonadaceae | 2 |
| Hyphomonadaceae | 2 |
| Rhodobacteraceae | 4 |
| Nocardiaceae | 3 |
| Propionibacteriaceae | 1 |
| Bradyrhizobiaceae | 11 |
| Myxococcaceae | 2 |
| Halomonadaceae | 1 |
| Idiomarinaceae | 1 |
| Alcaligenaceae | 5 |
| Bartonellaceae | 4 |
| Vibrionaceae | 12 |
| Mycobacteriaceae | 18 |
| Rhodospirillaceae | 4 |
| Colwelliaceae | 1 |
| Thermaceae | 2 |
| Desulfovibrionaceae | 4 |
| Spirochaetaceae | 3 |
| Cellulomonadaceae | 2 |
| Helicobacteraceae | 7 |
| Prochloraceae | 12 |
| Gloeobacteraceae | 1 |
| Mycoplasmataceae | 2 |
| Phyllobacteriaceae | 2 |
| Clostridiaceae | 12 |
| Alteromonadaceae | 1 |
| Dictyoglomaceae | 2 |
| Ectothiorhodospiraceae | 2 |
| Thermomicrobiaceae | 1 |
| Caulobacteraceae | 4 |
| Oceanospirillaceae | 1 |
| Staphylococcaceae | 1 |
| Neisseriaceae | 6 |
| Halanaerobiaceae | 1 |
| Comamonadaceae | 8 |
| Cyanothecaceae | 1 |
| Paenibacillaceae | 1 |
| Acidobacteriaceae | 1 |
| Xanthobacteraceae | 3 |
| Nostocaceae | 3 |
| Natranaerobiaceae | 1 |
| Heliobacteriaceae | 1 |
| Frankiaceae | 2 |
| Peptococcaceae | 4 |
| Beijerinckiaceae | 1 |
| Chlorobiaceae | 3 |
| Francisellaceae | 9 |
| Lachnospiraceae | 1 |
| Acaryochloridaceae | 1 |
| Yersiniaceae | 11 |
| Sphingomonadaceae | 4 |
| Psychromonadaceae | 1 |
| Nocardioidaceae | 1 |
| Zoogloeaceae | 1 |
| Microcoleaceae | 1 |
| Erythrobacteraceae | 1 |
| Syntrophomonadaceae | 1 |
| Chromatiaceae | 1 |
| Pelagibacteraceae | 1 |
| Pelobacteraceae | 1 |
| Nitrosomonadaceae | 3 |
| Thermoanaerobacteriaceae | 3 |
| Methylophilaceae | 1 |
| Hahellaceae | 1 |
| Dehalococcoidacea | 3 |
| Rhobocyclaceae | 2 |
| Bacteroidaceae | 5 |
| Alcanivoraceae | 1 |
| Methylococcaceae | 1 |
| Desulfobulbaceae | 1 |
| Microbacteriaceae | 2 |
| Planctomycetaceae | 1 |
| Aquificaceae | 2 |
| Porphyromonadaceae | 2 |
| Brucellaceae | 10 |
| Campylobacteraceae | 8 |
| Xanthomonadaceae | 12 |
| Merismopediaceae | 1 |
| Fusobacteriaceae | 1 |
| Hafniaceae | 1 |
| Morganellaceae | 2 |
| Nocardiopsaceae | 1 |
| Pseudonocardiaceae | 1 |
| Peptostreptococcaceae | 1 |
| Leptospiraceae | 4 |
| Aeromonadaceae | 2 |
| Erwiniaceae | 7 |
| Streptomycetaceae | 5 |
| Microcystaceae | 1 |
| Tannerellaceae | 1 |
| Acetobacteraceae | 1 |
| Poaceae | 3 |
| Geobacteraceae | 4 |
| Solanaceae | 2 |
| Pichiaceae | 1 |
| Cercopithecidae | 2 |
| Hominidae | 4 |
| Muridae | 4 |
| Dictyostelids | 1 |
| Bovidae | 3 |
| Physaraceae | 1 |
| Streptococcaceae | 2 |
| Amaranthaceae | 1 |
| Linderniaceae | 3 |
| Chromobacteriaceae | 2 |
| Cystobacterineae | 3 |
| Cellvibrionaceae | 2 |
| Hydrogenophilaceae | 1 |
| Parachlamydiaceae | 1 |
| Syntrophaceae | 1 |
| NA | 2 |
