# Supplementary material for: Excavating the functionally crucial active-site residues of the DXS protein of Bacillus subtilis by exploring its closest homologues
Source: J Genet Eng Biotechnol. 2020 Nov 26;18:76. doi: 10.1186/s43141-020-00087-x (PMC7691408; doi:10.1186/s43141-020-00087-x)
Supplement: Supplementary file 2 — Additional file 2: Figure S1. Multiple sequence alignment of the constructed dataset against the reference structure 6OUW. The alignment is constructed through ClustalO algorithm and is parsed against 6OUW through Espript3 server. The red shading indicates the sequence conservation. [file 43141_2020_87_MOESM2_ESM.pdf]

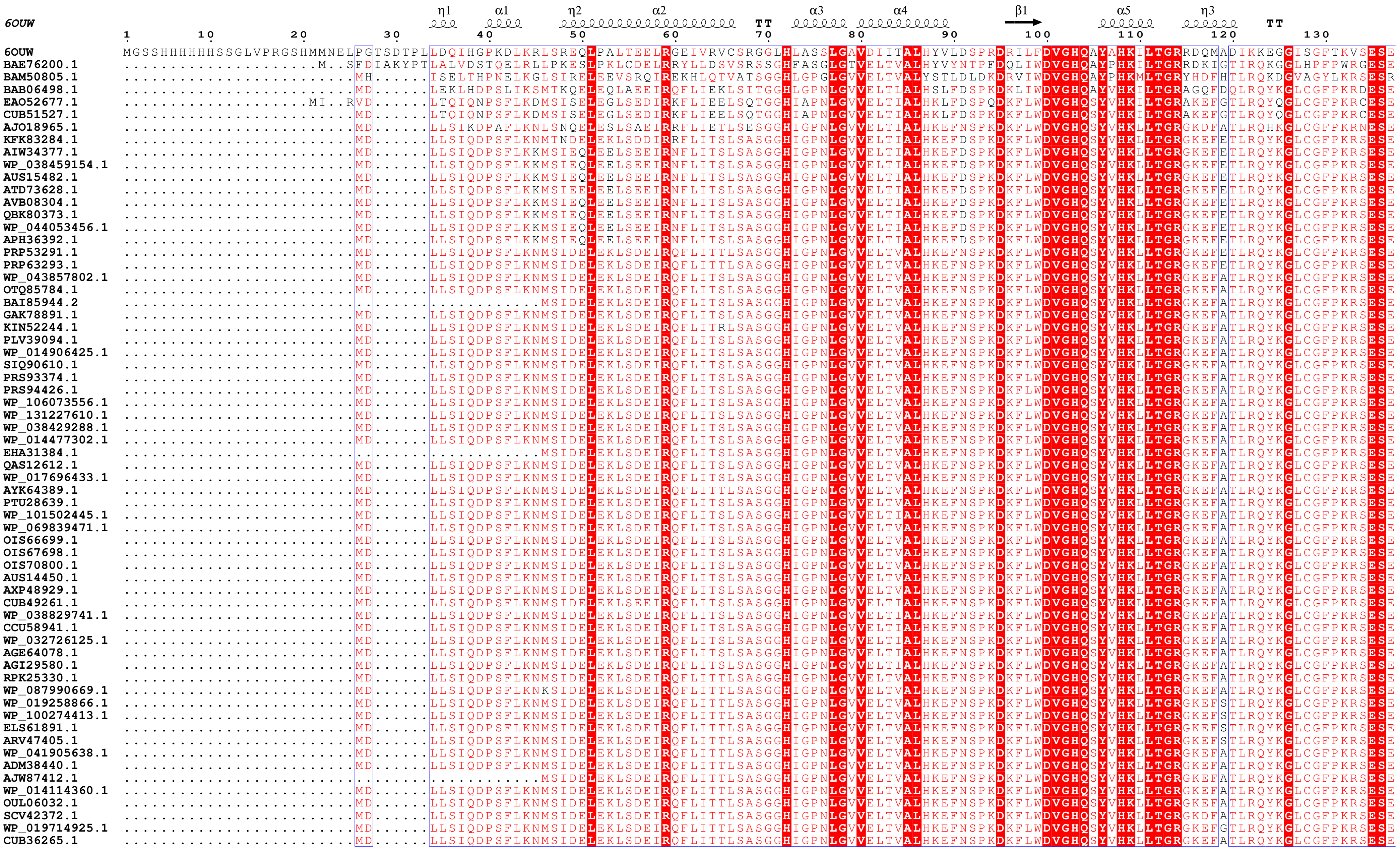
6OUW
η1
α1
η2
α2
TT
α3
α4
β1
α5
η3
TT
1
10
20
30
40
50
60
70
80
90
100
110
120
130
6OUW
BAE76200.1
BAM50805.1
BAB06498.1
EAO52677.1
CUB51527.1
AJO18965.1
KFK83284.1
AIW34377.1
WP_038459154.1
AUS15482.1
ATD73628.1
AVB08304.1
QBK80373.1
WP_044053456.1
APH36392.1
PRP53291.1
PRP63293.1
WP_043857802.1
OTQ85784.1
BAI85944.2
GAK78891.1
KIN52244.1
PLV39094.1
WP_014906425.1
SIQ90610.1
PRS93374.1
PRS94426.1
WP_106073556.1
WP_131227610.1
WP_038429288.1
WP_014477302.1
EHA31384.1
QAS12612.1
WP_017696433.1
AYK64389.1
PTU28639.1
WP_101502445.1
WP_069839471.1
OIS66699.1
OIS67698.1
OIS70800.1
AUS14450.1
AXP48929.1
CUB49261.1
WP_038829741.1
CCU58941.1
WP_032726125.1
AGE64078.1
AGI29580.1
RPK25330.1
WP_087990669.1
WP_019258866.1
WP_100274413.1
ELS61891.1
ARV47405.1
WP_041905638.1
ADM38440.1
AJW87412.1
WP_014114360.1
OUL06032.1
SCV42372.1
WP_019714925.1
CUB36265.1

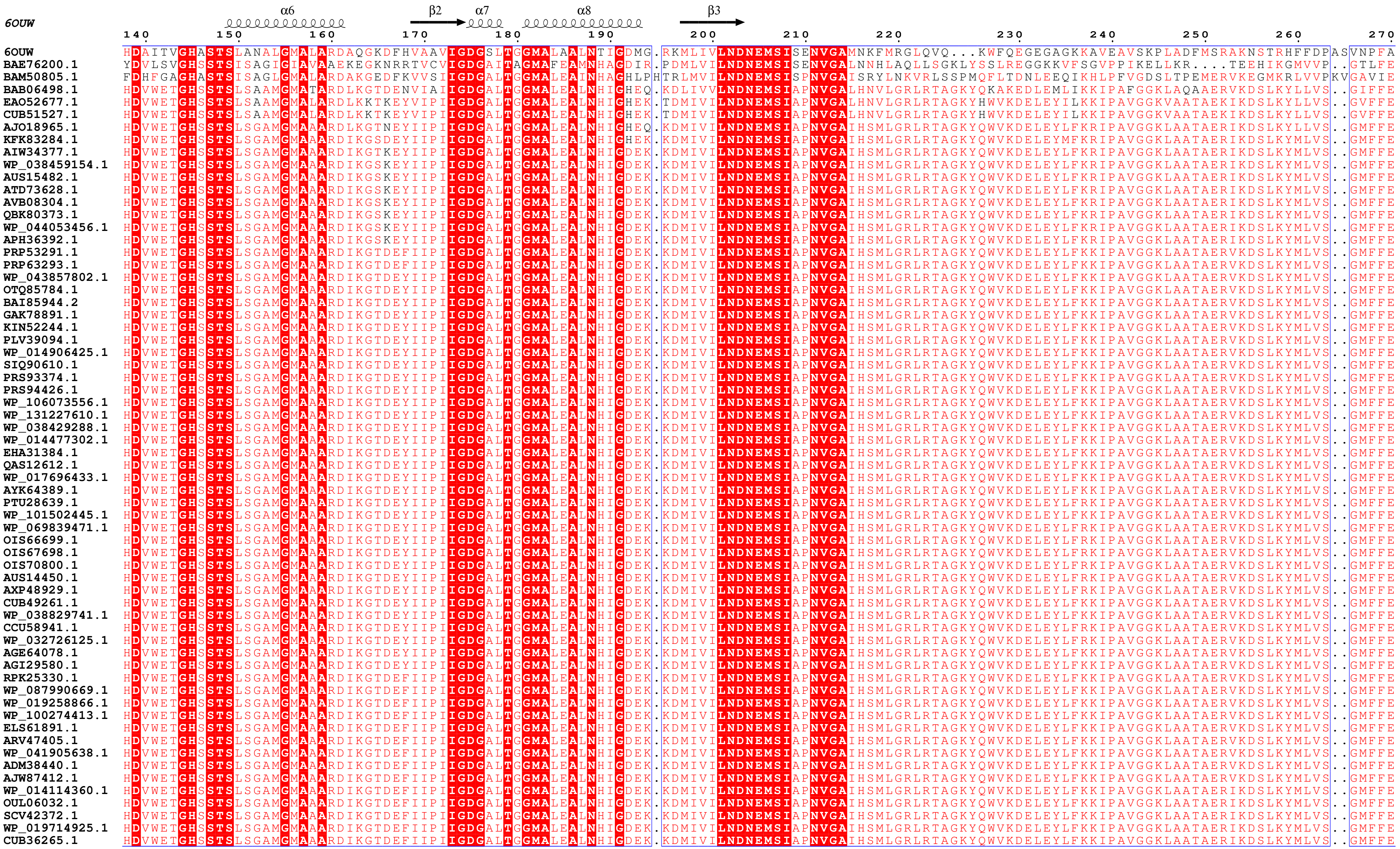
6OUW
α6
β2
α7
α8
β3
140 150 160 170 180 190 200 210 220 230 240 250 260 270
6OUW HDAITVGHASTSLANALGMALARDAQGKDFHVAAVIGDGSLTGGMALAALNTIGDMG.RKMLIVLNDNEMSISENVGAMNKFMRGLQVQ...KWFQEGEGAGKKAVEAVSKPLADFMSRAKNSTRHFFDPASVNPFA
BAE76200.1 YDVLSVGHSSTSISAGIGIAVAAEKEGKNRRTVCVIGDGAITAGMAFEAMNHAGDIR.PDMLVILNDNEMSISENVGALNNHLAQLLSGKLYSSLREGGKKVFSGVPPIKELLKR....TEEHIKGMVVP..GTLFE
BAM50805.1 FDHFGAGHASTSISAGLGMALARDAKGEDFKVVSIIGDGALTGGMALEAINHAGHLPHTRLMVILNDNEMSISPNVGAISRYLNKVRLSSPMQFLTDNLEEQIKHLPFVGDSLTPEMERVKEGMKRLVVPKVGAVIE
BAB06498.1 HDVWETGHSSTSLSAAMGMATARDLKGTDENVIAIIGDGALTGGMALEALNHIGHEQ.KDLIVVLNDNEMSIAPNVGALHNVLGRLRTAGKYQKAKEDLEMLIKKIPAFGGKLAQAAERVKDSLKYLLVS..GIFFE
EAO52677.1 HDVWETGHSSTSLSAAMGMALARDLKKTKEYVIPIIGDGALTGGMALEALNHIGHEK.TDMIVILNDNEMSIAPNVGALHNVLGRLRTAGKYHWVKDELEYILKKIPAVGGKVAATAEKIKDSLKYLLVS..GVFFE
CUB51527.1 HDVWETGHSSTSLSAAMGMALARDLKKTKEYVIPIIGDGALTGGMALEALNHIGHEK.TDMIVILNDNEMSIAPNVGALHNVLGRLRTAGKYHWVKDELEYILKKIPAVGGKVAATAEKIKDSLKYLLVS..GVFFE
AJO18965.1 HDVWETGHSSTSLSGAMGMAAARDLKGTNEYIIPIIGDGALTGGMALEALNHIGHEQ.KDMIVILNDNEMSIAPNVGAIHSMLGRLRTAGKYQWVKDELEYLFKRIPAVGGKLAATAERIKDSLKYLLVS..GMFFE
KFK83284.1 HDVWETGHSSTSLSGAMGMAAARDIKGSDEYIIPIIGDGALTGGMALEALNHIGHEK.KDMIVILNDNEMSIAPNVGAIHSMLGRLRTAGKYQWVKDELEYMFKRIPAVGGKLAATAERIKDSLKYMLVS..GMFFE
AIW34377.1 HDVWETGHSSTSLSGAMGMAAARDIKGTKEYIIPIIGDGALTGGMALEALNHIGDEK.KDMIVILNDNEMSIAPNVGAIHSMLGRLRTAGKYQWVKDELEYLFKRIPAVGGKLAATAERIKDSLKYMLVS..GMFFE
WP_038459154.1 HDVWETGHSSTSLSGAMGMAAARDIKGSKEYIIPIIGDGALTGGMALEALNHIGDEK.KDMIVILNDNEMSIAPNVGAIHSMLGRLRTAGKYQWVKDELEYLFKRIPAVGGKLAATAERIKDSLKYMLVS..GMFFE
AUS15482.1 HDVWETGHSSTSLSGAMGMAAARDIKGSKEYIIPIIGDGALTGGMALEALNHIGDEK.KDMIVILNDNEMSIAPNVGAIHSMLGRLRTAGKYQWVKDELEYLFKRIPAVGGKLAATAERIKDSLKYMLVS..GMFFE
ATD73628.1 HDVWETGHSSTSLSGAMGMAAARDIKGSKEYIIPIIGDGALTGGMALEALNHIGDEK.KDMIVILNDNEMSIAPNVGAIHSMLGRLRTAGKYQWVKDELEYLFKRIPAVGGKLAATAERIKDSLKYMLVS..GMFFE
AVB08304.1 HDVWETGHSSTSLSGAMGMAAARDIKGSKEYIIPIIGDGALTGGMALEALNHIGDEK.KDMIVILNDNEMSIAPNVGAIHSMLGRLRTAGKYQWVKDELEYLFKRIPAVGGKLAATAERIKDSLKYMLVS..GMFFE
QBK80373.1 HDVWETGHSSTSLSGAMGMAAARDIKGSKEYIIPIIGDGALTGGMALEALNHIGDEK.KDMIVILNDNEMSIAPNVGAIHSMLGRLRTAGKYQWVKDELEYLFKRIPAVGGKLAATAERIKDSLKYMLVS..GMFFE
WP_044053456.1 HDVWETGHSSTSLSGAMGMAAARDIKGSKEYIIPIIGDGALTGGMALEALNHIGDEK.KDMIVILNDNEMSIAPNVGAIHSMLGRLRTAGKYQWVKDELEYLFKRIPAVGGKLAATAERIKDSLKYMLVS..GMFFE
APH36392.1 HDVWETGHSSTSLSGAMGMAAARDIKGSKEYIIPIIGDGALTGGMALEALNHIGDEK.KDMIVILNDNEMSIAPNVGAIHSMLGRLRTAGKYQWVKDELEYLFKRIPAVGGKLAATAERIKDSLKYMLVS..GMFFE
PRP53291.1 HDVWETGHSSTSLSGAMGMAAARDIKGTDEFIIPIIGDGALTGGMALEALNHIGDEK.KDMIVILNDNEMSIAPNVGAIHSMLGRLRTAGKYQWVKDELEYLFKRIPAVGGKLAATAERVKDSLKYMLVS..GMFFE
PRP63293.1 HDVWETGHSSTSLSGAMGMAAARDIKGTDEFIIPIIGDGALTGGMALEALNHIGDEK.KDMIVILNDNEMSIAPNVGAIHSMLGRLRTAGKYQWVKDELEYLFKKIPAVGGKLAATAERVKDSLKYMLVS..GMFFE
WP_043857802.1 HDVWETGHSSTSLSGAMGMAAARDIKGTDEYIIPIIGDGALTGGMALEALNHIGDEK.KDMIVILNDNEMSIAPNVGAIHSMLGRLRTAGKYQWVKDELEYLFKKIPAVGGKLAATAERVKDSLKYMLVS..GMFFE
OTQ85784.1 HDVWETGHSSTSLSGAMGMAAARDIKGTDEYIIPIIGDGALTGGMALEALNHIGDEK.KDMIVILNDNEMSIAPNVGAIHSMLGRLRTAGKYQWVKDELEYLFKKIPAVGGKLAATAERVKDSLKYMLVS..GMFFE
BAI85944.2 HDVWETGHSSTSLSGAMGMAAARDIKGTDEYIIPIIGDGALTGGMALEALNHIGDEK.KDMIVILNDNEMSIAPNVGAIHSMLGRLRTAGKYQWVKDELEYLFKKIPAVGGKLAATAERVKDSLKYMLVS..GMFFE
GAK78891.1 HDVWETGHSSTSLSGAMGMAAARDIKGTDEYIIPIIGDGALTGGMALEALNHIGDEK.KDMIVILNDNEMSIAPNVGAIHSMLGRLRTAGKYQWVKDELEYLFKKIPAVGGKLAATAERVKDSLKYMLVS..GMFFE
KIN52244.1 HDVWETGHSSTSLSGAMGMAAARDIKGTDEYIIPIIGDGALTGGMALEALNHIGDEK.KDMIVILNDNEMSIAPNVGAIHSMLGRLRTAGKYQWVKDELEYLFKKIPAVGGKLAATAERVKDSLKYMLVS..GMFFE
PLV39094.1 HDVWETGHSSTSLSGAMGMAAARDIKGTDEYIIPIIGDGALTGGMALEALNHIGDEK.KDMIVILNDNEMSIAPNVGAIHSMLGRLRTAGKYQWVKDELEYLFKKIPAVGGKLAATAERVKDSLKYMLVS..GMFFE
WP_014906425.1 HDVWETGHSSTSLSGAMGMAAARDIKGTDEYIIPIIGDGALTGGMALEALNHIGDEK.KDMIVILNDNEMSIAPNVGAIHSMLGRLRTAGKYQWVKDELEYLFKKIPAVGGKLAATAERVKDSLKYMLVS..GMFFE
SIQ90610.1 HDVWETGHSSTSLSGAMGMAAARDIKGTDEYIIPIIGDGALTGGMALEALNHIGDEK.KDMIVILNDNEMSIAPNVGAIHSMLGRLRTAGKYQWVKDELEYLFKKIPAVGGKLAATAERVKDSLKYMLVS..GMFFE
PRS93374.1 HDVWETGHSSTSLSGAMGMAAARDIKGTDEYIIPIIGDGALTGGMALEALNHIGDEK.KDMIVILNDNEMSIAPNVGAIHSMLGRLRTAGKYQWVKDELEYLFKKIPAVGGKLAATAERVKDSLKYMLVS..GMFFE
PRS94426.1 HDVWETGHSSTSLSGAMGMAAARDIKGTDEYIIPIIGDGALTGGMALEALNHIGDEK.KDMIVILNDNEMSIAPNVGAIHSMLGRLRTAGKYQWVKDELEYLFKKIPAVGGKLAATAERVKDSLKYMLVS..GMFFE
WP_106073556.1 HDVWETGHSSTSLSGAMGMAAARDIKGTDEYIIPIIGDGALTGGMALEALNHIGDEK.KDMIVILNDNEMSIAPNVGAIHSMLGRLRTAGKYQWVKDELEYLFKKIPAVGGKLAATAERVKDSLKYMLVS..GMFFE
WP_131227610.1 HDVWETGHSSTSLSGAMGMAAARDIKGTDEYIIPIIGDGALTGGMALEALNHIGDEK.KDMIVILNDNEMSIAPNVGAIHSMLGRLRTAGKYQWVKDELEYLFKKIPAVGGKLAATAERVKDSLKYMLVS..GMFFE
WP_038429288.1 HDVWETGHSSTSLSGAMGMAAARDIKGTDEYIIPIIGDGALTGGMALEALNHIGDEK.KDMIVILNDNEMSIAPNVGAIHSMLGRLRTAGKYQWVKDELEYLFKKIPAVGGKLAATAERVKDSLKYMLVS..GMFFE
WP_014477302.1 HDVWETGHSSTSLSGAMGMAAARDIKGTDEYIIPIIGDGALTGGMALEALNHIGDEK.KDMIVILNDNEMSIAPNVGAIHSMLGRLRTAGKYQWVKDELEYLFKKIPAVGGKLAATAERVKDSLKYMLVS..GMFFE
EHA31384.1 HDVWETGHSSTSLSGAMGMAAARDIKGTDEYIIPIIGDGALTGGMALEALNHIGDEK.KDMIVILNDNEMSIAPNVGAIHSMLGRLRTAGKYQWVKDELEYLFKKIPAVGGKLAATAERVKDSLKYMLVS..GMFFE
QAS12612.1 HDVWETGHSSTSLSGAMGMAAARDIKGTDEYIIPIIGDGALTGGMALEALNHIGDEK.KDMIVILNDNEMSIAPNVGAIHSMLGRLRTAGKYQWVKDELEYLFKKIPAVGGKLAATAERVKDSLKYMLVS..GMFFE
WP_017696433.1 HDVWETGHSSTSLSGAMGMAAARDIKGTDEYIIPIIGDGALTGGMALEALNHIGDEK.KDMIVILNDNEMSIAPNVGAIHSMLGRLRTAGKYQWVKDELEYLFKKIPAVGGKLAATAERVKDSLKYMLVS..GMFFE
AYK64389.1 HDVWETGHSSTSLSGAMGMAAARDIKGTDEYIIPIIGDGALTGGMALEALNHIGDEK.KDMIVILNDNEMSIAPNVGAIHSMLGRLRTAGKYQWVKDELEYLFKKIPAVGGKLAATAERVKDSLKYMLVS..GMFFE
PTU28639.1 HDVWETGHSSTSLSGAMGMAAARDIKGTDEYIIPIIGDGALTGGMALEALNHIGDEK.KDMIVILNDNEMSIAPNVGAIHSMLGRLRTAGKYQWVKDELEYLFRKIPAVGGKLAATAERVKDSLKYMLVS..GMFFE
WP_101502445.1 HDVWETGHSSTSLSGAMGMAAARDIKGTDEYIIPIIGDGALTGGMALEALNHIGDEK.KDMIVILNDNEMSIAPNVGAIHSMLGRLRTAGKYQWVKDELEYLFKKIPAVGGKLAATAERVKDSLKYMLVS..GMFFE
WP_069839471.1 HDVWETGHSSTSLSGAMGMAAARDIKGTDEYIIPIIGDGALTGGMALEALNHIGDEK.KDMIVILNDNEMSIAPNVGAIHSMLGRLRTAGKYQWVKDELEYLFRKIPAVGGKLAATAERVKDSLKYMLVS..GMFFE
OIS66699.1 HDVWETGHSSTSLSGAMGMAAARDIKGTDEYIIPIIGDGALTGGMALEALNHIGDEK.KDMIVILNDNEMSIAPNVGAIHSMLGRLRTAGKYQWVKDELEYLFRKIPAVGGKLAATAERVKDSLKYMLVS..GMFFE
OIS67698.1 HDVWETGHSSTSLSGAMGMAAARDIKGTDEYIIPIIGDGALTGGMALEALNHIGDEK.KDMIVILNDNEMSIAPNVGAIHSMLGRLRTAGKYQWVKDELEYLFRKIPAVGGKLAATAERVKDSLKYMLVS..GMFFE
OIS70800.1 HDVWETGHSSTSLSGAMGMAAARDIKGTDEYIIPIIGDGALTGGMALEALNHIGDEK.KDMIVILNDNEMSIAPNVGAIHSMLGRLRTAGKYQWVKDELEYLFRKIPAVGGKLAATAERVKDSLKYMLVS..GMFFE
AUS14450.1 HDVWETGHSSTSLSGAMGMAAARDIKGTDEYIIPIIGDGALTGGMALEALNHIGDEK.KDMIVILNDNEMSIAPNVGAIHSMLGRLRTAGKYQWVKDELEYLFRKIPAVGGKLAATAERVKDSLKYMLVS..GMFFE
AXP48929.1 HDVWETGHSSTSLSGAMGMAAARDIKGTDEYIIPIIGDGALTGGMALEALNHIGDEK.KDMIVILNDNEMSIAPNVGAIHSMLGRLRTAGKYQWVKDELEYLFRKIPAVGGKLAATAERVKDSLKYMLVS..GMFFE
CUB49261.1 HDVWETGHSSTSLSGAMGMAAARDIKGTDEYIIPIIGDGALTGGMALEALNHIGDEK.KDMIVILNDNEMSIAPNVGAIHSMLGRLRTAGKYQWVKDELEYLFKKIPAVGGKLAATAERVKDSLKYMLVS..GMFFE
WP_038829741.1 HDVWETGHSSTSLSGAMGMAAARDIKGTDEYIIPIIGDGALTGGMALEALNHIGDEK.KDMIVILNDNEMSIAPNVGAIHSMLGRLRTAGKYQWVKDELEYLFKKIPAVGGKLAATAERVKDSLKYMLVS..GMFFE
CCU58941.1 HDVWETGHSSTSLSGAMGMAAARDIKGTDEYIIPIIGDGALTGGMALEALNHIGDEK.KDMIVILNDNEMSIAPNVGAIHSMLGRLRTAGKYQWVKDELEYLFRKIPAVGGKLAATAERVKDSLKYMLVS..GMFFE
WP_032726125.1 HDVWETGHSSTSLSGAMGMAAARDIKGTDEYIIPIIGDGALTGGMALEALNHIGDEK.KDMIVILNDNEMSIAPNVGAIHSMLGRLRTAGKYQWVKDELEYLFRKIPAVGGKLAATAERVKDSLKYMLVS..GMFFE
AGE64078.1 HDVWETGHSSTSLSGAMGMAAARDIKGTDEYIIPIIGDGALTGGMALEALNHIGDEK.KDMIVILNDNEMSIAPNVGAIHSMLGRLRTAGKYQWVKDELEYLFKKIPAVGGKLAATAERVKDSLKYMLVS..GMFFE
AGI29580.1 HDVWETGHSSTSLSGAMGMAAARDIKGTDEYIIPIIGDGALTGGMALEALNHIGDEK.KDMIVILNDNEMSIAPNVGAIHSMLGRLRTAGKYQWVKDELEYLFKKIPAVGGKLAATAERVKDSLKYMLVS..GMFFE
RPK25330.1 HDVWETGHSSTSLSGAMGMAAARDIKGTDEFIIPIIGDGALTGGMALEALNHIGDEK.KDMIVILNDNEMSIAPNVGAIHSMLGRLRTAGKYQWVKDELEYLFKKIPAVGGKLAATAERVKDSLKYMLVS..GMFFE
WP_087990669.1 HDVWETGHSSTSLSGAMGMAAARDIKGTDEFIIPIIGDGALTGGMALEALNHIGDEK.KDMIVILNDNEMSIAPNVGAIHSMLGRLRTAGKYQWVKDELEYLFKKIPAVGGKLAATAERVKDSLKYMLVS..GMFFE
WP_019258866.1 HDVWETGHSSTSLSGAMGMAAARDIKGTDEFIIPIIGDGALTGGMALEALNHIGDEK.KDMIVILNDNEMSIAPNVGAIHSMLGRLRTAGKYQWVKDELEYLFKKIPAVGGKLAATAERVKDSLKYMLVS..GMFFE
WP_100274413.1 HDVWETGHSSTSLSGAMGMAAARDIKGTDEFIIPIIGDGALTGGMALEALNHIGDEK.KDMIVILNDNEMSIAPNVGAIHSMLGRLRTAGKYQWVKDELEYLFKKIPAVGGKLAATAERVKDSLKYMLVS..GMFFE
ELS61891.1 HDVWETGHSSTSLSGAMGMAAARDIKGTDEFIIPIIGDGALTGGMALEALNHIGDEK.KDMIVILNDNEMSIAPNVGAIHSMLGRLRTAGKYQWVKDELEYLFKKIPAVGGKLAATAERVKDSLKYMLVS..GMFFE
ARV47405.1 HDVWETGHSSTSLSGAMGMAAARDIKGTDEFIIPIIGDGALTGGMALEALNHIGDEK.KDMIVILNDNEMSIAPNVGAIHSMLGRLRTAGKYQWVKDELEYLFKKIPAVGGKLAATAERVKDSLKYMLVS..GMFFE
WP_041905638.1 HDVWETGHSSTSLSGAMGMAAARDIKGTDEFIIPIIGDGALTGGMALEALNHIGDEK.KDMIVILNDNEMSIAPNVGAIHSMLGRLRTAGKYQWVKDELEYLFKKIPAVGGKLAATAERVKDSLKYMLVS..GMFFE
ADM38440.1 HDVWETGHSSTSLSGAMGMAAARDIKGTDEFIIPIIGDGALTGGMALEALNHIGDEK.KDMIVILNDNEMSIAPNVGAIHSMLGRLRTAGKYQWVKDELEYLFKKIPAVGGKLAATAERVKDSLKYMLVS..GMFFE
AJW87412.1 HDVWETGHSSTSLSGAMGMAAARDIKGTDEFIIPIIGDGALTGGMALEALNHIGDEK.KDMIVILNDNEMSIAPNVGAIHSMLGRLRTAGKYQWVKDELEYLFKKIPAVGGKLAATAERVKDSLKYMLVS..GMFFE
WP_014114360.1 HDVWETGHSSTSLSGAMGMAAARDIKGTDEFIIPIIGDGALTGGMALEALNHIGDEK.KDMIVILNDNEMSIAPNVGAIHSMLGRLRTAGKYQWVKDELEYLFKKIPAVGGKLAATAERIKDSLKYMLVS..GMFFE
OUL06032.1 HDVWETGHSSTSLSGAMGMAAARDIKGTDEFIIPIIGDGALTGGMALEALNHIGDEK.KDMIVILNDNEMSIAPNVGAIHSMLGRLRTAGKYQWVKDELEYLFKKIPAVGGKLAATAERIKDSLKYMLVS..GMFFE
SCV42372.1 HDVWETGHSSTSLSGAMGMAAARDIKGTDEFIIPIIGDGALTGGMALEALNHIGDEK.KDMIVILNDNEMSIAPNVGAIHSMLGRLRTAGKYQWVKDELEYLFKKIPAVGGKLAATAERIKDSLKYMLVS..GMFFE
WP_019714925.1 HDVWETGHSSTSLSGAMGMAAARDIKGTDEFIIPIIGDGALTGGMALEALNHIGDEK.KDMIVILNDNEMSIAPNVGAIHSMLGRLRTAGKYQWVKDELEYLFKKIPAVGGKLAATAERIKDSLKYMLVS..GMFFE
CUB36265.1 HDVWETGHSSTSLSGAMGMAAARDIKGTDEFIIPIIGDGALTGGMALEALNHIGDEK.KDMIVILNDNEMSIAPNVGAIHSMLGRLRTAGKYQWVKDELEYLFKKIPAVGGKLAATAERIKDSLKYMLVS..GMFFE

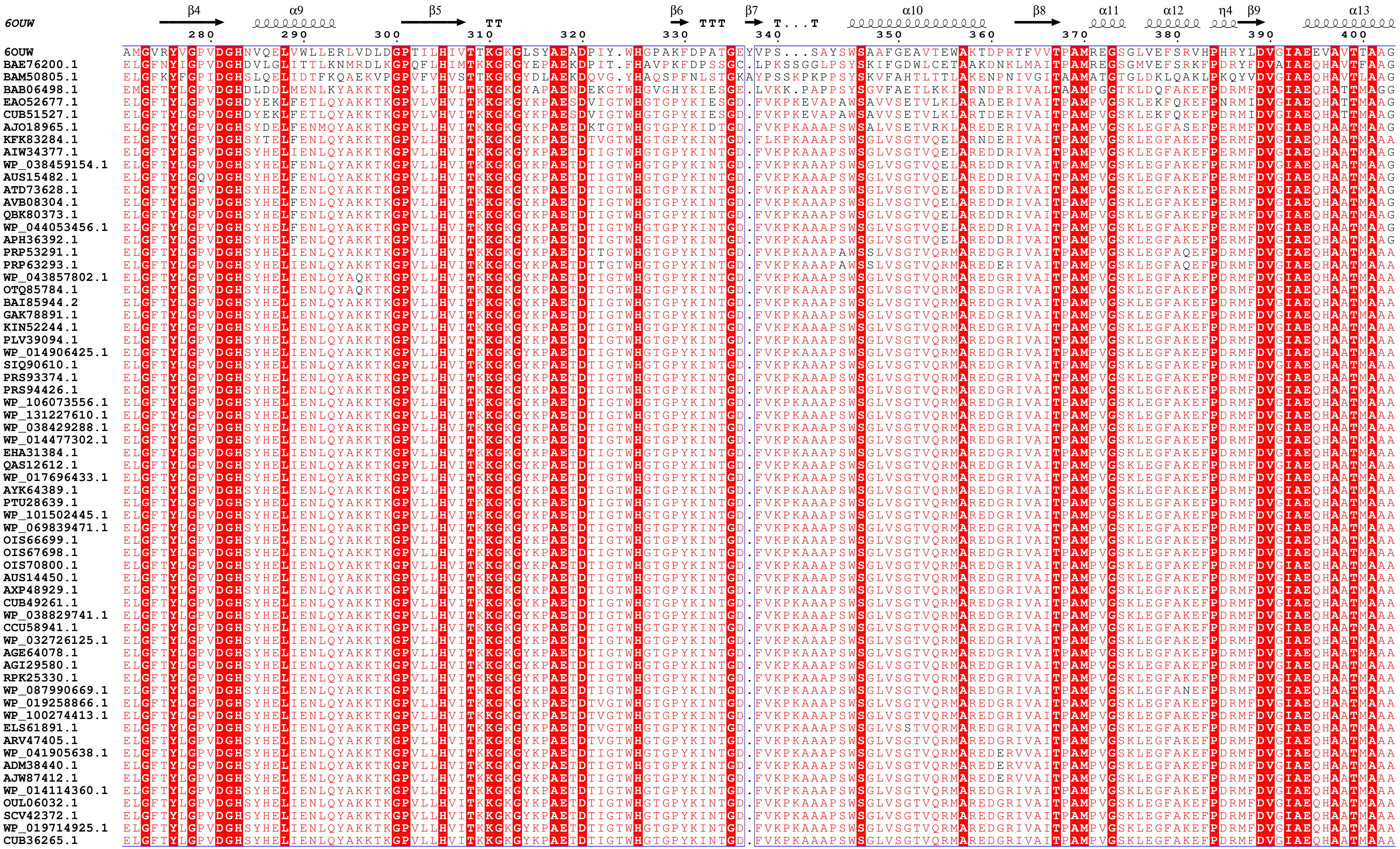
6OUW
β4
α9
β5
TT
β6
TTT
β7
T...T
α10
β8
α11
α12
η4
β9
α13
280
290
300
310
320
330
340
350
360
370
380
390
400
6OUW
BAE76200.1
BAM50805.1
BAB06498.1
EAO52677.1
CUB51527.1
AJO18965.1
KFK83284.1
AIW34377.1
WP_038459154.1
AUS15482.1
ATD73628.1
AVB08304.1
QBK80373.1
WP_044053456.1
APH36392.1
PRP53291.1
PRP63293.1
WP_043857802.1
OTQ85784.1
BAI85944.2
GAK78891.1
KIN52244.1
PLV39094.1
WP_014906425.1
SIQ90610.1
PRS93374.1
PRS94426.1
WP_106073556.1
WP_131227610.1
WP_038429288.1
WP_014477302.1
EHA31384.1
QAS12612.1
WP_017696433.1
AYK64389.1
PTU28639.1
WP_101502445.1
WP_069839471.1
OIS66699.1
OIS67698.1
OIS70800.1
AUS14450.1
AXP48929.1
CUB49261.1
WP_038829741.1
CCU58941.1
WP_032726125.1
AGE64078.1
AGI29580.1
RPK25330.1
WP_087990669.1
WP_019258866.1
WP_100274413.1
ELS61891.1
ARV47405.1
WP_041905638.1
ADM38440.1
AJW87412.1
WP_014114360.1
OUL06032.1
SCV42372.1
WP_019714925.1
CUB36265.1

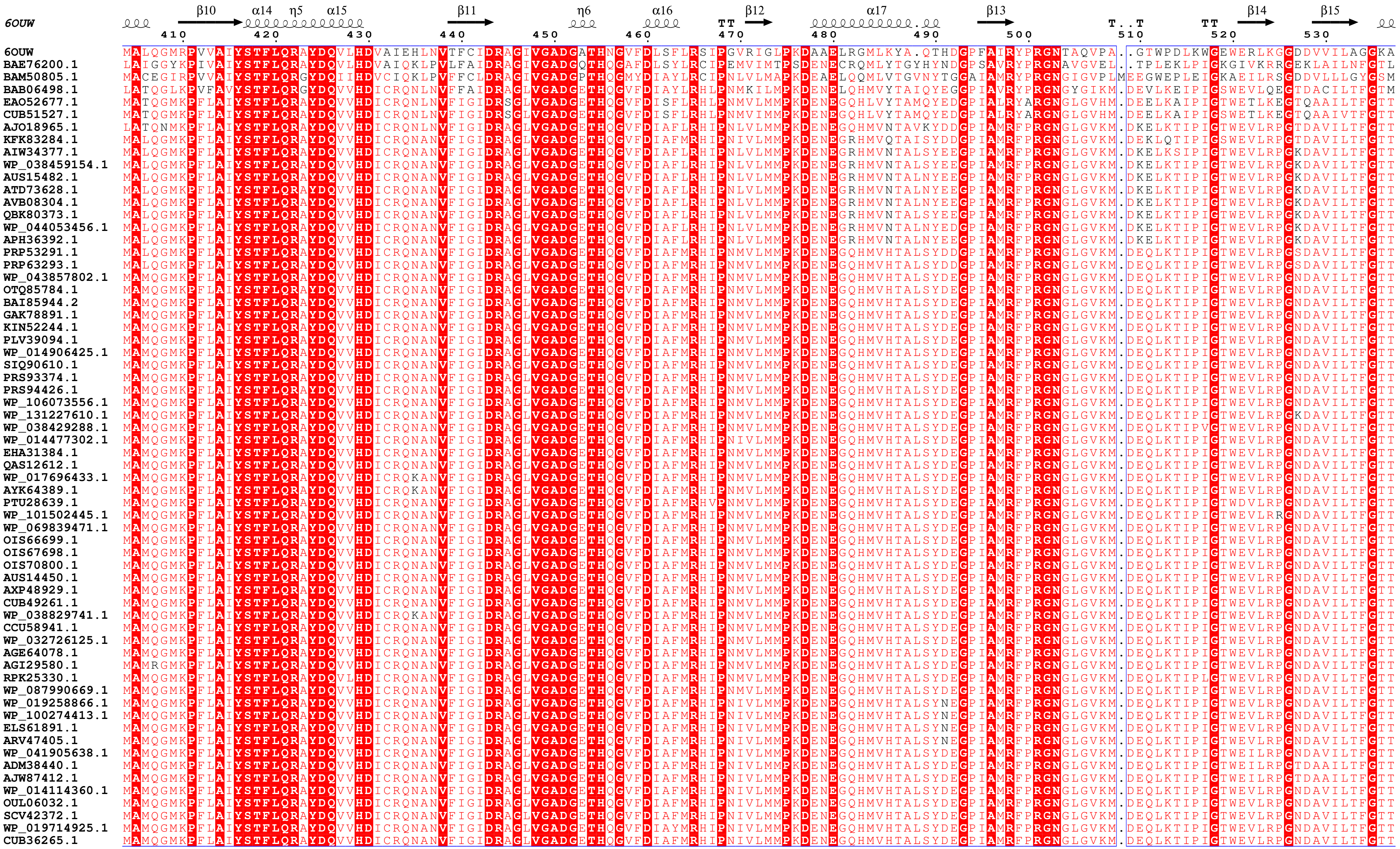
6OUW
β10
α14
η5
α15
β11
η6
α16
TT
β12
α17
β13
T..T
TT
β14
β15
410
420
430
440
450
460
470
480
490
500
510
520
530
6OUW
BAE76200.1
BAM50805.1
BAB06498.1
EAO52677.1
CUB51527.1
AJO18965.1
KFK83284.1
AIW34377.1
WP_038459154.1
AUS15482.1
ATD73628.1
AVB08304.1
QBK80373.1
WP_044053456.1
APH36392.1
PRP53291.1
PRP63293.1
WP_043857802.1
OTQ85784.1
BAI85944.2
GAK78891.1
KIN52244.1
PLV39094.1
WP_014906425.1
SIQ90610.1
PRS93374.1
PRS94426.1
WP_106073556.1
WP_131227610.1
WP_038429288.1
WP_014477302.1
EHA31384.1
QAS12612.1
WP_017696433.1
AYK64389.1
PTU28639.1
WP_101502445.1
WP_069839471.1
OIS66699.1
OIS67698.1
OIS70800.1
AUS14450.1
AXP48929.1
CUB49261.1
WP_038829741.1
CCU58941.1
WP_032726125.1
AGE64078.1
AGI29580.1
RPK25330.1
WP_087990669.1
WP_019258866.1
WP_100274413.1
ELS61891.1
ARV47405.1
WP_041905638.1
ADM38440.1
AJW87412.1
WP_014114360.1
OUL06032.1
SCV42372.1
WP_019714925.1
CUB36265.1

```
                   α18        TT     β16    β17      α19         β18          α20             β19           α21         α22
6OUW               540       550        560       570       580       590       600       610       620       630       640       650
6OUW               LDYALKAAEDLPG....VGVVNARFVKPLDEEMLREVGGRARALITVEDNTVVGGFGGAVLEALNSMNL.HPTVRVLGIPDEFQEHATAESVHARAGIDAPAIRTVLAELGVDVPIEV.......
BAE76200.1         MPEAAKVAESL.....NATLVDMRFVKPLDEALILEMAASHEALVTVEENAIMGGAGSGVNEVLMAHRK.PVPVLNIGLPDFFIPQGTQEEMRAELGLDAAGMEAKIKAWLA.............
BAM50805.1         VYPALQTAELLHEHGIEATVVNARFVKPLDTELILPLAERIGKVVTMEEGCLMGGFGSAVAEALMDNNV.LVPLKRLGVPDILVDHATPEQSTVDLGLTPAQMAQNIMASLFKTETESVVAPGVS
BAB06498.1         IPVAEQASKELSQQGYSIRLINARSVKPLDEAMLHEIAKSGRPVLTLEETAVQGSFGSAVLEFFHDHGYHNVVTQRMGIPDRFIEHGSVSELLEEIGLTSSQVANQLSKLLPRKQKRA.......
EAO52677.1         IPMAMEAAERLEKAGVSVKVVNARFIKPMDEAYLHDLLGKNIPILTIEEACLIGGFGTGVVEFASENGYHSALVERMGIPDRFIEHGSVTKLLEEIGLTTDAVVDRIHTMIPSKLKRA.......
CUB51527.1         IPMAMEAAERLEKAGVSVKVVNARFIKPMDEAYLHDLLGKNIPILTIEEACLIGGFGTGVVEFASENGYHSALVERMGIPDRFIEHGSVTKLLEEIGLTTDAVVDRIHTMIPSKQKRA.......
AJO18965.1         IPMALAAAEELQKEGRSVRVVNARFIKPLDENMLMEILNEGLPILTIEEAVLQGGFGSSILEYAHEHQSYSPIIDRMGIPDQFIEHGSVAKLLEEIGMTKEDVIRRIRLLTPVKTHKGIGS....
KFK83284.1         IEMALEAAEELQKEGRSVRVVNARFIKPIDKKMMKDILGEGLPILTIEEAVLEGGFGSSILEFAQEQGMYHTPIDRMGIPDQFIEHGSVTALLEEIGLTKQQAANRLRLLTPRKTHKGIGS....
AIW34377.1         IEMALEAAEELQKEGLSVRVVNARFIKPIDKQMMKAILNEGLPILTIEEAVLEGGFGSTILEFAHDLGMYHTPIDRMGIPDRFIEHGSVTALLEEIGLTKAEVMNRIKLLMPPKTHKGIGS....
WP_038459154.1     IEMALEAAEELQKEGLSVRVVNARFIKPIDQQMMKAILNEGLPILTIEEAVLEGGFGSTILEFAHNLGMYHTPIDRMGIPDRFIEHGSVTALLEEIGLTKAEVMNRIKLLMPPKTHKGIGS....
AUS15482.1         IEMALEAAEELQKEGLSVRVVNARFIKPIDKQMMKAILNEGLPILTIEEAVLEGGFGSTILEFAHDLGMYHTPIDRMGIPDRFIEHGSVTALLEEIGLTKAEVMNRIKLLMPPKTHKGIGS....
ATD73628.1         IEMALEAAEELQKEGLSVRVVNARFIKPIDKQMMKAILNEGLPILTIEEAVLEGGFGSTILEFAHDLGMYHTPIDRMGIPDRFIEHGSVTALLEEIGLTKAEVMNRIKLLMPPKTHKGIGS....
AVB08304.1         IEMALEAAEELQKEGLSVRVVNARFIKPIDKQMMKAILNEGLPILTIEEAVLEGGFGSTILEFAHDLGMYHTPIDRMGIPDRFIEHGSVTALLEEIGLTKAEVMNRIKLLMPPKTHKGIGS....
QBK80373.1         IEMALEAAEELQKEGLSVRVVNARFIKPIDKQMMKAILNEGLPILTIEEAVLEGGFGSTILEYAHDLGMYHTPIDRMGIPDRFIEHGSVTALLEEIGLTKAEVMNRIKLLMPPKTHKGIGS....
WP_044053456.1     IEMALEAAEELQKEGLSVRVVNARFIKPIDKQMMKAILNEGLPILTIEEAVLEGGFGSTILEYAHDLGMYHTPIDRMGIPDRFIEHGSVTALLEEIGLTKAEVMNRIKLLMPPKTHKGIGS....
APH36392.1         IEMALEAAEELQKEGLSVRVVNARFIKPIDKQMMKAILNEGLPILTIEEAVLEGGFGSTILEYAHDLGMYHTPIDRMGIPDRFIEHGSVTALLEEIGLTKAEVMNRIKLLMPPKTHKGIGS....
PRP53291.1         IEMALEAAEELQKEGLSVRVVNARFIKPIDEKMMKDILNEGLPILTIEEAVLEGGFGSAVLEFAHDQGMYHTPIDRMGIPDRFIEHGSVTALLEEIGLTKQQVADRIRLLAPPKTHKGIGS....
PRP63293.1         IEMALEAAEELQKEGLSVRVVNARFIKPIDEKMMKDILNEGLPILTIEEAVLEGGFGSAVLEFAHDQGMYHTPIDRMGIPDRFIEHGSVTALLEEIGLTKQQVADRIRLLAPPKTHKGIGS....
WP_043857802.1     IEMAIEAAEELQKEGLSVRVVNARFIKPIDEKMMKSILKEGLPILTIEEAVLEGGFGSSILEFAHDQGKYHTPIDRMGIPDRFIEHGSVTALLKEIGLTKQQVANRIRLLMPPKTHKGIGS....
OTQ85784.1         IEMAIEAAEELQKEGLSVRVVNARFIKPIDEKMMKSILKEGLPILTIEEAVLEGGFGSSILEFAHDQGEYHTPIDRMGIPDRFIEHGSVTALLKEIGLTKQQVANRIRLLMPPKTHKGIGS....
BAI85944.2         IEMAIEAAEELQKEGLSVRVVNARFIKPIDEKMMKSILKEGLPILTIEEAVLEGGFGSSILEFAHDQGEYHTPIDRMGIPDRFIEHGSVTALLKEIGLTKQQVANRIRLLMPSKTHKGIGS....
GAK78891.1         IEMAIEAAEELQKEGLSVRVVNARFIKPIDEKMMKSILKEGLPILTIEEAVLEGGFGSSILEFAHDQGEYHTPIDRMGIPDRFIEHGSVTALLKEIGLTKQQVANRIRLLMPSKTHKGIGS....
KIN52244.1         IEMAIEAAEELQKEGLSVRVVNARFIKPIDEKMMKSILKEGLPILTIEEAVLEGGFGSSILEFAHDQGEYHTPIDRMGIPDRFIEHGSVTALLKEIGLTKQQVANRIRLLMPPKTHKGIGS....
PLV39094.1         IEMAIEAAEELQKEGLSVRVVNARFIKPIDEKMMKSILKEGLPILTIEEAVLEGGFGSSILEFAHDQGEYHTPIDRMGIPDRFIEHGSVTALLKEIGLTKQQVANRIRLLMPPKTHKGIGS....
WP_014906425.1     IEMAIEAAEELQKEGLSVRVVNARFIKPIDEKMMKSILKEGLPILTIEEAVLEGGFGSSILEFAHDQGKYHTPIDRMGIPDRFIEHGSVTALLEEIGLTKQQVANRIRLLMPPKTHKGIGS....
SIQ90610.1         IEMAIEAAEELQKEGLSVRVVNARFIKPIDEKMMKSILKEGLPILTIEEAVLEGGFGSSILEFAHDQGQYHTPIDRMGIPDRFIEHGSVTALLEEIGLTKQQVANRIRLLMPPKTHKGIGS....
PRS93374.1         IEMAIEAAEELQKEGLSVRVVNARFIKPIDEKMMKSILKEGLPILTIEEAVLEGGFGSSILEFAHDQGEYHTPIDRMGIPDRFIEHGSVAALLEEIGLTKQQVANRIRLLMPPKTHKGIGS....
PRS94426.1         IEMAIEAAEELQKEGLSVRVVNARFIKPIDEKMMKSILKEGLPILTIEEAVLEGGFGSSILEFAHDQGEYHTPIDRMGIPDRFIEHGSVAALLEEIGLTKQQVANRIRLLMPPKTHKGIGS....
WP_106073556.1     IEMAIEAAEELQKEGLSVRVVNARFIKPIDEKMMKSILKEGLPILTIEEAVLEGGFGSSILEFAHDQGEYHTPIDRMGIPDRFIEHGSVAALLEEIGLTKQQVANRIRLLMPPKTHKGIGS....
WP_131227610.1     IEMAIEAAEELQKEGLSVRVVNARFIKPIDEKMMKSILKEGLPILTIEEAVLEGGFGSSILEFAHDQGEYHTPIDRMGIPDRFIEHGSVTALLEEIGLTKQQVANRIRLLMPPKTHKGIGS....
WP_038429288.1     IEMAIEAAEELQKEGLSVRVVNARFIKPIDEKMMKSILKEGLPILTIEEAVLEGGFGSSILEFAHDQGEYHTPIDRMGIPDRFIEHGSVTALLEEIGLTKQQVANRIRLLMPPKTHKGIGS....
WP_014477302.1     IEMAIEAAEELQKEGLSVRVVNARFIKPIDEKMMKSILKEGLPILTIEEAVLEGGFGSSILEFAHDQGEYHTPIDRMGIPDRFIEHGSVTALLEEIGLTKQQVANRIRLLMPPKTHKGIGS....
EHA31384.1         IEMAIEAAEELQKEGLSVRVVNARFIKPIDEKMMKSILKEGLPILTIEEAVLEGGFGSSILEFAHDQGEYHTPIDRMGIPDRFIEHGSVTALLEEIGLTKQQVANRIRLLMPPKTHKGIGS....
QAS12612.1         IEMAIEAAEELQKEGLSVRVVNARFIKPIDEKMMKSILKEGLPILTIEEAVLEGGFGSSILEFAHDQGEYHTPIDRMGIPDRFIEHGSVTALLEEIGLTKQQVANRIRLLMPPKTHKGIGS....
WP_017696433.1     IEMAIEAAEELQKEGLSVRVVNARFIKPIDEKMMKSILKEGLPILTIEEAVLEGGFGSSILEFAHDQGEYHTPIDRMGIPDRFIEHGSVTALLEEIGLTKQQVANRIRLLMSPKTHKGIGS....
AYK64389.1         IEMAIEAAEELQKEGLSVRVVNARFIKPIDEKMMKSILKEGLPILTIEEAVLEGGFGSSILEFAHDQGEYHTPIDRMGIPDRFIEHGSVTALLEEIGLTKQQVANRIRLLMSPKTHKGIGS....
PTU28639.1         IEMAIEAAEELQKEGLSVRVVNARFIKPIDEKMMKSILKEGLPILTIEEAVLEGGFGSSILEFAHDQGEYHTPIDRMGIPDRFIEHGSVTALLEEIGLTKQQVANRIRLLMPPKTHKGIGS....
WP_101502445.1     IEMAIEAAEELQKEGLSVRVVNARFIKPIDEKMMKSILKEGLPILTIEEAVLEGGFGSSILEFAHDQGEYHTPIDRMGIPDRFIEHGSVTALLEEIGLTKQQVANRIRLLMPPKTHKGIGS....
WP_069839471.1     IEMAIEAAEELQKEGLSVRVVNARFIKPIDEKMMKSILKEGLPILTIEEAVLEGGFGSSILEFAHDQGEYHTPIDRMGIPDRFIEHGSVTALLEEIGLTKQQVANRIRLLMPPKTHKGIGS....
OIS66699.1         IEMAIEAAEELQKEGLSVRVVNARFIKPIDEKMMKSILKEGLPILTIEEAVLEGGFGSSILEFAHDQGEYHTPIDRMGIPDRFIEHGSVTALLEEIGLTKQQVANRIRLLMPPKTHKGIGS....
OIS67698.1         IEMAIEAAEELQKEGLSVRVVNARFIKPIDEKMMKSILKEGLPILTIEEAVLEGGFGSSILEFAHDQGEYHTPIDRMGIPDRFIEHGSVTALLEEIGLTKQQVANRIRLLMPPKTHKGIGS....
OIS70800.1         IEMAIEAAEELQKEGLSVRVVNARFIKPIDEKMMKSILKEGLPILTIEEAVLEGGFGSSILEFAHDQGEYHTPIDRMGIPDRFIEHGSVTALLEEIGLTKQQVANRIRLLMPPKTHKGIGS....
AUS14450.1         IEMAIEAAEELQKEGLSVRVVNARFIKPIDEKMMKSILKEGLPILTIEEAVLEGGFGSSILEFAHDQGEYHTPIDRMGIPDRFIEHGSVTALLEEIGLTKQQVANRIRLLMPPKTHKGIGS....
AXP48929.1         IEMAIEAAEELQKEGLSVRVVNARFIKPIDEKMMKSILKEGLPILTIEEAVLEGGFGSSILEFAHDQGEYHTPIDRMGIPDRFIEHGSVTALLEEIGLTKQQVANRIRLLMPPKTHKGIGS....
CUB49261.1         IEMAIEAAEELQKEGLSVRVVNARFIKPIDEKMMKSILKEGLPILTIEEAVLEGGFGSSILEFAHDQGEYHTPIDRMGIPDRFIEHGSVTALLEEIGLTKQQVANRIRLLMPPKTHKGIGS....
WP_038829741.1     IEMAIEAAEELQKEGLSVRVVNARFIKPIDEKMMKSILKEGLPILTIEEAVLEGGFGSSILEFAHDQGEYHTPIDRMGIPDRFIEHGSVTALLEEIGLTKQQVANRIRLLMPPKTHKGIGS....
CCU58941.1         IEMAIEAAEELQKEGLSVRVVNARFIKPIDEKMMKSILKEGLPILTIEEAVLEGGFGSSILEFAHDQGEYHTPIDRMGIPDRFIEHGSVTALLEEIGLTKQQVANRIRLLMPPKTHKGIGS....
WP_032726125.1     IEMAIEAAEELQKEGLSVRVVNARFIKPIDEKMMKSILKEGLPILTIEEAVLEGGFGSSILEFAHDQGEYHTPIDRMGIPDRFIEHGSVTALLEEIGLTKQQVANRIRLLMPPKTHKGIGS....
AGE64078.1         IEMAIEAAEELQKEGLSVRVVNARFIKPIDEKMMKSILKEGLPILTIEEAVLEGGFGSSILEFAHDQGEYHTPIDRMGIPDRFIEHGSVTALLEEIGLTKQQVANRIRLLMPPKTHKGIGS....
AGI29580.1         IEMAIEAAEELQKEGLSVRVVNARFIKPIDEKMMKSILKEGLPILTIEEAVLEGGFGSSILEFAHDQGEYHTPIDRMGIPDRFIEHGSVTALLEEIGLTKQQVANRIRLLMPPKTHKGIGS....
RPK25330.1         IEMALEAAEELQKEGLSVRVVNARFIKPIDEKMMKDILKEGLPILTIEEAVLEGGFGSSILEFAHDQGEYHTPIDRMGIPDRFIEHGNVTALLEEIGLTKQQTANRIRLMMPPKTHKGIGS....
WP_087990669.1     IEMALEAAEELQKEGLSVRVVNARFIKPMDEKMMKDILKEGLPILTIEEAVLEGGFGSSILEFAHDQGEFLTPIDRMGIPDRFIEHGSVTALLEEIGLTKQQAANRIRLLMPPKTHKGIGS....
WP_019258866.1     IEMALEAAEELQKEGLSVRVVNARFIKPIDEKMMKDILKEGLPILTIEEAVLEGGFGSSILEFAHDQGEYLSPIDRMGIPDRFIEHGSVTALLEEIGLTKQQVANRIRLLMPPKTHKGIGS....
WP_100274413.1     IEMALEAAEELQKEGLSVRVVNARFIKPIDEKMMKDILKEGLPILTIEEAVLEGGFGSSILEFAHDQGEYLTPIDRMGIPDRFIEHGSVTALLEEIGLTKQQVADRIRLLMPPKTHKGIGS....
ELS61891.1         IEMALEAAEELQKEGLSVRVVNARFIKPIDEKMMKDILKEGLPILTIEEAVLEGGFGSSILEFAHDQGEYLTPIDRMGIPDRFIEHGSVTALLEEIGLTKQQAADRIRLLMPPKTHKGIGS....
ARV47405.1         IEMALEAAEELQKEGLSVRVVNARFIKPIDEKMMKDILKEGLPILTIEEAVLEGGFGSSILEFAHDQGEYLTPIDRMGIPDRFIEHGSVTALLEEIGLTKQQAADRIRLLMPPKTHKGIGS....
WP_041905638.1     IEMALEAAEELQKEGLSVRVVNARFIKPIDEKMMKGILKEGLPILTIEEAVLEGGFGSSILEFAHDQGEYLTPIDRMGIPDRFIEHGSVTALLEEIGLTKQQVANRIRLLMPPKTHKGIGS....
ADM38440.1         IEMALEAAEELQKEGLSVRVVNARFIKPIDEKMMKGILKEGLPILTIEEAVLEGGFGSSILEFAHDQGEYLTPIDRMGIPDRFIEHGSVTALLEEIGLTKQQVANRIRLLMPPKTHKGIGS....
AJW87412.1         IEMALEAAEELQKEGLSVRVVNARFIKPIDEKMMKGILKEGLPILTIEEAVLEGGFGSSILEFAHDQGEYLTPIDRMGIPDRFIEHGSVTALLEEIGLTKQQVANRIRLLMPPKTHKGIGS....
WP_014114360.1     IEMTLEAAEELQKEGLSVRVVNARFIKPIDEKMMKSILKEGLPILTIEEAVLEGGFGSSILEFAHDQGEYHTPIDRMGIPDRFIEHGSVTALLEEIGLTKQQVANRIRLLMPPKTHKGIGS....
OUL06032.1         IEMALEAAEELQKEGLSVRVVNARFIKPIDEKMMKSILKEGLPILTIEEAVLEGGFGSSILEFAHDQGEYHTPIDRMGIPDRFIEHGSVTALLEEIGLTKQQVANRIRLLMPPKTHKGIGS....
SCV42372.1         IEMAFEAAEELQKEGLSVRVVNARFIKPIDEKMMKGILKEGLPILTIEEAVLEGGFGSSILEFAHDQGEYLTPIDRMGIPDRFIEHGSVTALLEEIGLTKQQVANRIRLLMPPKTHKGIGS....
WP_019714925.1     IEMALEAAEELQKEGLSVRVVNARFIKPIDEKMMKGILKEGLPILTIEEAVLEGGFGSSILEFAHDQGEYLTPIDRMGIPDRFIEHGSVTALLEEIGLTKQQVANRIRLLMPPKTHKGIGS....
CUB36265.1         IEMALEAAEELQKEGLSVRVVNARFIKPIDEKMMKGILKEGLPILTIEEAVLEGGFGSSILEFAHDQGEYLTPIDRMGIPDRFIEHGSVTALLEEIGLTKQQVANRIRLLMPPKTHKGIGS....
```
